# Concurrent RB1 Loss and *BRCA* Deficiency Predicts Enhanced Immunologic Response and Long-term Survival in Tubo-ovarian High-grade Serous Carcinoma

**DOI:** 10.1158/1078-0432.CCR-23-3552

**Published:** 2024-06-05

**Authors:** Flurina A.M. Saner, Kazuaki Takahashi, Timothy Budden, Ahwan Pandey, Dinuka Ariyaratne, Tibor A. Zwimpfer, Nicola S. Meagher, Sian Fereday, Laura Twomey, Kathleen I. Pishas, Therese Hoang, Adelyn Bolithon, Nadia Traficante, for the Australian Ovarian Cancer Study Group, Kathryn Alsop, Elizabeth L. Christie, Eun-Young Kang, Gregg S. Nelson, Prafull Ghatage, Cheng-Han Lee, Marjorie J. Riggan, Jennifer Alsop, Matthias W. Beckmann, Jessica Boros, Alison H. Brand, Angela Brooks-Wilson, Michael E. Carney, Penny Coulson, Madeleine Courtney-Brooks, Kara L. Cushing-Haugen, Cezary Cybulski, Mona A. El-Bahrawy, Esther Elishaev, Ramona Erber, Simon A. Gayther, Aleksandra Gentry-Maharaj, C. Blake Gilks, Paul R. Harnett, Holly R. Harris, Arndt Hartmann, Alexander Hein, Joy Hendley, Brenda Y. Hernandez, Anna Jakubowska, Mercedes Jimenez-Linan, Michael E. Jones, Scott H. Kaufmann, Catherine J. Kennedy, Tomasz Kluz, Jennifer M. Koziak, Björg Kristjansdottir, Nhu D. Le, Marcin Lener, Jenny Lester, Jan Lubiński, Constantina Mateoiu, Sandra Orsulic, Matthias Ruebner, Minouk J. Schoemaker, Mitul Shah, Raghwa Sharma, Mark E. Sherman, Yurii B. Shvetsov, T. Rinda Soong, Helen Steed, Paniti Sukumvanich, Aline Talhouk, Sarah E. Taylor, Robert A. Vierkant, Chen Wang, Martin Widschwendter, Lynne R. Wilkens, Stacey J. Winham, Michael S. Anglesio, Andrew Berchuck, James D. Brenton, Ian Campbell, Linda S. Cook, Jennifer A. Doherty, Peter A. Fasching, Renée T. Fortner, Marc T. Goodman, Jacek Gronwald, David G. Huntsman, Beth Y. Karlan, Linda E. Kelemen, Usha Menon, Francesmary Modugno, Paul D.P. Pharoah, Joellen M. Schildkraut, Karin Sundfeldt, Anthony J. Swerdlow, Ellen L. Goode, Anna DeFazio, Martin Köbel, Susan J. Ramus, David D.L. Bowtell, Dale W. Garsed

**Affiliations:** 1 Peter MacCallum Cancer Centre, Melbourne, Australia.; 2 Department of Obstetrics and Gynecology, Bern University Hospital and University of Bern, Bern, Switzerland.; 3 Department of Obstetrics and Gynecology, The Jikei University School of Medicine, Tokyo, Japan.; 4 School of Clinical Medicine, UNSW Medicine and Health, University of NSW Sydney, Sydney, Australia.; 5 Skin Cancer and Ageing Lab, Cancer Research United Kingdom Manchester Institute, The University of Manchester, Manchester, United Kingdom.; 6 The Daffodil Centre, The University of Sydney, A Joint Venture with Cancer Council New South Wales, Sydney, Australia.; 7 Sir Peter MacCallum Department of Oncology, The University of Melbourne, Parkville, Australia.; 8 Adult Cancer Program, Lowy Cancer Research Centre, University of NSW Sydney, Sydney, Australia.; 9 Department of Pathology and Laboratory Medicine, Foothills Medical Center, University of Calgary, Calgary, Canada.; 10 Division of Gynecologic Oncology, Department of Oncology, Cumming School of Medicine, University of Calgary, Calgary, Canada.; 11 Department of Laboratory Medicine and Pathology, University of Alberta, Edmonton, Canada.; 12 Division of Gynecologic Oncology, Department of Obstetrics and Gynecology, Duke University Medical Center, Durham, North Carolina.; 13 Department of Oncology, Centre for Cancer Genetic Epidemiology, University of Cambridge, Cambridge, United Kingdom.; 14 Department of Gynecology and Obstetrics, Comprehensive Cancer Center Erlangen-EMN, Friedrich-Alexander University Erlangen-Nuremberg, University Hospital Erlangen, Erlangen, Germany.; 15 Centre for Cancer Research, The Westmead Institute for Medical Research, Sydney, Australia.; 16 Department of Gynaecological Oncology, Westmead Hospital, Sydney, Australia.; 17 The University of Sydney, Sydney, Australia.; 18 Canada’s Michael Smith Genome Sciences Centre, Vancouver, Canada.; 19 Department of Obstetrics and Gynecology, John A. Burns School of Medicine, University of Hawaii, Honolulu, Hawaii.; 20 Division of Genetics and Epidemiology, The Institute of Cancer Research, London, United Kingdom.; 21 Department of Obstetrics, Gynecology and Reproductive Sciences, University of Pittsburgh School of Medicine, Pittsburgh, Pennsylvania.; 22 Program in Epidemiology, Division of Public Health Sciences, Fred Hutchinson Cancer Center, Seattle, Washington.; 23 Department of Genetics and Pathology, International Hereditary Cancer Center, Pomeranian Medical University, Szczecin, Poland.; 24 Department of Metabolism, Digestion and Reproduction, Imperial College London, Hammersmith Hospital, London, United Kingdom.; 25 Department of Pathology, University of Pittsburgh School of Medicine, Pittsburgh, Pennsylvania.; 26 Institute of Pathology, Comprehensive Cancer Center Erlangen-EMN, Friedrich-Alexander University Erlangen-Nuremberg, University Hospital Erlangen, Erlangen, Germany.; 27 Center for Bioinformatics and Functional Genomics and the Cedars Sinai Genomics Core, Cedars-Sinai Medical Center, Los Angeles, California.; 28 MRC Clinical Trials Unit, Institute of Clinical Trials and Methodology, University College London, London, United Kingdom.; 29 Department of Women’s Cancer, Elizabeth Garrett Anderson Institute for Women’s Health, University College London, London, United Kingdom.; 30 Department of Pathology and Laboratory Medicine, University of British Columbia, Vancouver, Canada.; 31 Crown Princess Mary Cancer Centre, Westmead Hospital, Sydney, Australia.; 32 Department of Epidemiology, University of Washington, Seattle, Washington.; 33 University of Hawaii Cancer Center, Honolulu, Hawaii.; 34 Independent Laboratory of Molecular Biology and Genetic Diagnostics, Pomeranian Medical University, Szczecin, Poland.; 35 Department of Histopathology, Addenbrooke’s Hospital, Cambridge, United Kingdom.; 36 Division of Oncology Research, Department of Oncology, Mayo Clinic, Rochester, Minnesota.; 37 Department of Gynecology and Obstetrics, Gynecology Oncology and Obstetrics, Institute of Medical Sciences, Medical College of Rzeszow University, Rzeszów, Poland.; 38 Alberta Health Services-Cancer Care, Calgary, Canada.; 39 Department of Obstetrics and Gynecology, Institute of Clinical Sciences, Sahlgrenska Center for Cancer Research, University of Gothenburg, Gothenburg, Sweden.; 40 Cancer Control Research, BC Cancer Agency, Vancouver, Canada.; 41 Department of Genetics and Pathology, International Hereditary Cancer Center, Pomeranian Medical University in Szczecin, Szczecin, Poland.; 42 Department of Obstetrics and Gynecology, David Geffen School of Medicine, University of California at Los Angeles, Los Angeles, California.; 43 Department of Pathology, University of Gothenburg, Gothenburg, Sweden.; 44 Tissue Pathology and Diagnostic Oncology, Westmead Hospital, Sydney, Australia.; 45 Department of Health Sciences Research, Mayo Clinic, Jacksonville, Florida.; 46 Division of Gynecologic Oncology, Department of Obstetrics and Gynecology, University of Alberta, Edmonton, Canada.; 47 Section of Gynecologic Oncology Surgery, North Zone, Alberta Health Services, Edmonton, Canada.; 48 British Columbia’s Gynecological Cancer Research Team (OVCARE), BC Cancer, and Vancouver General Hospital, University of British Columbia, Vancouver, Canada.; 49 Department of Obstetrics and Gynecology, University of British Columbia, Vancouver, Canada.; 50 Department of Quantitative Health Sciences, Division of Clinical Trials and Biostatistics, Mayo Clinic, Rochester, Minnesota.; 51 Division of Computational Biology, Department of Quantitative Health Sciences, Mayo Clinic, Rochester, Minnesota.; 52 EUTOPS Institute, University of Innsbruck, Innsbruck, Austria.; 53 Cancer Research UK Cambridge Institute, University of Cambridge, Cambridge, United Kingdom.; 54 Department of Epidemiology, School of Public Health, University of Colorado, Aurora, Colorado.; 55 Community Health Sciences, University of Calgary, Calgary, Canada.; 56 Department of Population Health Sciences, Huntsman Cancer Institute, University of Utah, Salt Lake City, Utah.; 57 Division of Cancer Epidemiology, German Cancer Research Center (DKFZ), Heidelberg, Germany.; 58 Department of Research, Cancer Registry of Norway, Norwegian Institute of Public Health, Oslo, Norway.; 59 Cancer Prevention and Control Program, Cedars-Sinai Cancer, Cedars-Sinai Medical Center, Los Angeles, California.; 60 Department of Molecular Oncology, BC Cancer Research Centre, Vancouver, Canada.; 61 Division of Acute Disease Epidemiology, South Carolina Department of Health & Environmental Control, Columbia, South Carolina.; 62 Department of Epidemiology, University of Pittsburgh School of Public Health, Pittsburgh, Pennsylvania.; 63 Women’s Cancer Research Center, Magee-Womens Research Institute and Hillman Cancer Center, Pittsburgh, Pennsylvania.; 64 Department of Computational Biomedicine, Cedars-Sinai Medical Center, West Hollywood, California.; 65 Centre for Cancer Genetic Epidemiology, Department of Public Health and Primary Care, University of Cambridge, Cambridge, United Kingdom.; 66 Department of Epidemiology, Rollins School of Public Health, Emory University, Atlanta, Georgia.; 67 Division of Breast Cancer Research, The Institute of Cancer Research, London, United Kingdom.; 68 Division of Epidemiology, Department of Quantitative Health Sciences, Mayo Clinic, Rochester, Minnesota.

## Abstract

**Purpose:**

The purpose of this study was to evaluate RB1 expression and survival across ovarian carcinoma histotypes and how co-occurrence of *BRCA1* or *BRCA2* (*BRCA*) alterations and RB1 loss influences survival in tubo-ovarian high-grade serous carcinoma (HGSC).

**Experimental Design:**

RB1 protein expression was classified by immunohistochemistry in ovarian carcinomas of 7,436 patients from the Ovarian Tumor Tissue Analysis consortium. We examined RB1 expression and germline *BRCA* status in a subset of 1,134 HGSC, and related genotype to overall survival (OS), tumor-infiltrating CD8^+^ lymphocytes, and transcriptomic subtypes. Using CRISPR-Cas9, we deleted *RB1* in HGSC cells with and without *BRCA1* alterations to model co-loss with treatment response. We performed whole-genome and transcriptome data analyses on 126 patients with primary HGSC to characterize tumors with concurrent *BRCA* deficiency and *RB1* loss.

**Results:**

RB1 loss was associated with longer OS in HGSC but with poorer prognosis in endometrioid ovarian carcinoma. Patients with HGSC harboring both RB1 loss and pathogenic germline *BRCA* variants had superior OS compared with patients with either alteration alone, and their median OS was three times longer than those without pathogenic *BRCA* variants and retained RB1 expression (9.3 vs. 3.1 years). Enhanced sensitivity to cisplatin and paclitaxel was seen in *BRCA1*-altered cells with *RB1* knockout. Combined *RB1* loss and *BRCA* deficiency correlated with transcriptional markers of enhanced IFN response, cell-cycle deregulation, and reduced epithelial–mesenchymal transition. CD8^+^ lymphocytes were most prevalent in *BRCA*-deficient HGSC with co-loss of *RB1*.

**Conclusions:**

Co-occurrence of RB1 loss and *BRCA* deficiency was associated with exceptionally long survival in patients with HGSC, potentially due to better treatment response and immune stimulation.

**Translational Relevance**

Improved understanding of the gene alterations associated with homologous recombination deficiency (HRD) and drug sensitivity will enable better prognostication and treatment stratification in patients with HRD-prone cancers. In a large cohort of 7,436 patients with ovarian carcinoma, we found that tumor RB1 protein loss was most frequent (16.4%) in tubo-ovarian high-grade serous carcinoma and associated with longer overall survival. The positive effect of RB1 loss on survival was more pronounced in patients with co-occurring HRD gene alterations; most frequently germline *BRCA1* or *BRCA2* (*BRCA*) pathogenic variants. In contrast, patients with combined RB1 loss and homologous recombination proficiency exhibit a worse prognosis, suggesting the relationship between RB1 loss and survival is HRD-dependent. RB1 expression is assessable by an affordable and accessible immunohistochemistry assay and could be considered as a stratification factor, along with HRD tests, in future trials to determine whether it is predictive of response to chemotherapy and/or PARP inhibitors.

## Introduction

Despite a high response rate to primary treatment, the progressive development of acquired drug resistance is common in tubo-ovarian high-grade serous carcinoma (HGSC), a histotype that is associated with approximately 70% of ovarian cancer deaths (1). The frequent acquisition of resistance-conferring alterations in HGSC (2–4) suggests that the development of drug resistance may be inevitable when curative surgery is not achieved in these patients. Countering that view, however, is the observation that a small subset of patients with HGSC advanced disease experience an exceptional response to treatment, survive well beyond a median of 3.4 years (5), and in some cases, remain disease free (6, 7). Interest in studying long-term cancer survivors is growing, as they may assist in the discovery of prognostic biomarkers, novel treatments, and approaches to limit the development of resistance (8, 9).

Several clinical and molecular factors that influence treatment response and overall survival (OS) in HGSC have been described. Complete surgical debulking is associated with a more favorable outcome compared with patients left with residual disease (10–12). Molecular subtypes defined by distinct gene expression patterns in primary HGSC are associated with different outcomes (13), including the poor survival C1/mesenchymal subtype that is more often seen in patients for whom complete surgical tumor resection cannot be achieved (14–16). By contrast, the C2/immunoreactive subtype is typified by extensive infiltration of intraepithelial T cells (13), a feature known to be strongly associated with improved survival (17, 18). Tumors arising in individuals with germline or somatic alterations in *BRCA1* or *BRCA2* genes are typically more responsive to conventional chemotherapy and PARP inhibitors, whereas those tumors with intact homologous recombination (HR) DNA repair are more often resistant to treatment (19–21). Patients with germline *BRCA1* or *BRCA2* pathogenic variants (g*BRCA*var) show more favorable survival at 5 years post-diagnosis compared with those with wild-type germline *BRCA* genes (g*BRCA*wt), and those with germline *BRCA2* pathogenic variants retain a long-term (>10 years) survival advantage (22–24). Although deleterious alterations in *BRCA1*, *BRCA2*, and other genes involved in HR DNA repair are associated with a favorable response to treatment, these are not sufficient alone to confer long-term survival and a large proportion of such patients experience a typical disease trajectory. Differential outcomes in *BRCA*-driven HGSC can in part be ascribed to alternative splicing (25), retention of the wild-type *BRCA* allele in tumors (26), or the acquisition of reversion mutations (2, 3), all of which seem to limit the effectiveness of chemotherapy.

We previously characterized a small series of HGSC exceptional survivors and found that co-occurring loss-of-function alterations in both *BRCA* and *RB1* were associated with unusually favorable survival (7, 27). Disruption of the RB pathway is found in many cancer types but with variable impacts on patient outcomes. For example, co-loss of *RB1* and *BRCA* is associated with shorter survival in breast and prostate cancer, possibly due to lineage switching and resistance to hormonal therapy (28–30). A transcriptomic signature of RB1 loss was recently described to be associated with poor outcomes across cancer types (31). We have previously found that chromosomal breakage is the most common mechanism of *RB1* inactivation in HGSC (3), accounting for approximately 80% of all *RB1* alterations. In addition to its crucial role in cell cycle regulation, RB1 is involved in non-canonical functions in a context- and tissue-dependent manner (32–34), including HR-mediated DNA repair. Loss of RB1 expression in HGSC has been associated with a survival benefit (35), including in the context of abnormal block-like p16 staining (36).

Factors underlying the association of RB1 loss with improved outcomes in HGSC are unknown. Here, we contrast the pattern and clinical consequences of RB1 loss in HGSC with other epithelial ovarian cancer subtypes, investigate the relevance of co-occurring *BRCA1* or *BRCA2* alterations and RB1 loss in patients with HGSC, and explore the functional effects of combined *BRCA* and *RB1* impairment in HGSC cell lines.

## Materials and Methods

### Patient cohorts

The study population consisted of 7,436 patients diagnosed with invasive epithelial ovarian, peritoneal, or fallopian tube cancer from 20 studies or biobanks participating in the Ovarian Tumor Tissue Analysis (OTTA) consortium (ref. 37; Table 1; Supplementary Fig. S1). This study was conducted in accordance with the principles of Good Clinical Practice and the Declaration of Helsinki. Written informed consent or an institutional review board–approved waiver of consent was obtained at each site for patient recruitment, sample collection, and study protocols (Supplementary Table S1). Human investigations were performed after approval by local human research ethics committees/institutional review boards at each site and in accordance with an assurance filed with and approved by the US Department of Health and Human Services, where appropriate. Cases in this study were recruited before the widespread use of *BRCA* testing and PARP inhibitors (median year of diagnosis 2004, 25%–75% quartiles 2001–2007, 5%–95% percentiles 1993–2012, range, 1978–2016).

**Table 1.** Clinicopathologic characteristics and RB1 expression patterns across histotypes.

	HGSC		LGSC		MOC		ENOC		CCOC		Total		
	*n*	(%)	*n*	(%)	*N*	(%)	*N*	(%)	*n*	(%)	*N*	(%)	*P*
Patients													
Number (% of total)	5,009	(67)	224	(3)	409	(6)	1,033	(14)	761	(10)	7,436		
Age at diagnosis (years)													
Median	61		55		56		54		55		59		<0.0001^a^
Min–max	21–92		23–88		23–95		21–91		27–89		21–95		
1%–99% percentile	37–84		25–87		24–87		30–84		33–83		32–84		
FIGO stage													
I/II	894	(18)	67	(30)	310	(76)	805	(78)	567	(75)	2,643	(36)	<0.0001^b^
III/IV	3,841	(77)	137	(61)	57	(14)	147	(14)	168	(22)	4,350	(58)	
Unknown	274	(5)	20	(9)	42	(10)	81	(8)	26	(3)	443	(6)	
Residual disease													
Absent	1,023	(20)	73	(33)	162	(40)	461	(45)	352	(46.3)	2,071	(27.9)	<0.0001^b^
Present	1,488	(30)	52	(23)	21	(5)	41	(4)	78	(10.2)	1,680	(22.6)	
Unknown	2,498	(50)	99	(44)	226	(55)	531	(51)	331	(43.5)	3,685	(49.6)	
RB1 protein													
Loss	734	(15)	5	(2)	7	(2)	37	(4)	12	(2)	795	(11)	<0.0001^c^
Retained	3,748	(75)	176	(79)	319	(78)	871	(84)	655	(86)	5,769	(78)	
Subclonal loss	58	(1)	0	(0)	0	(0)	7	(1)	1	(0)	66	(1)	
Cytoplasmic	13	(0)	0	(0)	1	(0)	1	(0)	2	(0)	17	(0)	
Uninterpretable	456	(9)	43	(19)	82	(20)	117	(11)	91	(12)	789	(11)	

Abbreviations: CCOC, clear cell ovarian cancer; LGSC, low-grade serous carcinoma; MOC, mucinous ovarian cancer.

a Kruskal–Wallis test *P* values are reported, excluding cases with “unknown” information.

b *χ* ^2^ test *P* values are reported, excluding cases with “unknown” information.

c *χ* ^2^ test excluding cases with subclonal loss and cytoplasmic or uninterpretable RB1 protein expression.

Whole-genome sequence and matched transcriptome sequence data of primary HGSC tumors were available from 126 patients from the Multidisciplinary Ovarian Cancer Outcomes Group (MOCOG) study (ref. 27; Supplementary Fig. S1). This cohort consisted of 34 short-term survivors (OS < 2 years), 32 moderate-term survivors (OS ≥ 2 and <10 years), and 60 long-term survivors (OS ≥ 10 years) with advanced-stage (IIIC/IV) disease, enrolled in the Australian Ovarian Cancer Study (AOCS), the Gynaecological Oncology Biobank at Westmead Hospital (Sydney), or the Mayo Clinic Study.

### IHC staining and analysis of RB1 protein expression

RB1 protein expression was determined by IHC staining and scoring of tissue microarrays (TMA) from formalin-fixed, paraffin-embedded (FFPE) tumor samples, using our previously described protocol (7). Sections of 4 μm thickness of previously constructed TMAs, with each case represented by 1–3 cores (either 0.6, 1, or 2 mm in diameter), were shipped to a central IHC laboratory at the University of Calgary (Alberta, Canada). Detailed information on the anatomic site of the tissue microarray source is not available for every sample; however, for the OTTA studies for which this information is available, most cases (86%) were sampled from the adnexal tubo-ovarian tumor. FFPE samples on slides were subjected to heat-induced antigen retrieval with Target Retrieval Solution high on the DAKO Omnis platform (Agilent Technologies, Santa Clara, CA) and then incubated with anti-RB1 (Retinoblastoma Gene Protein) mouse mAb (Leica, Clone 13A10, Novocastra: #NCL-L-RB-358) at a 1:100 dilution. Staining was visualized using 3,3′-diaminobenzidine.

Samples were scored as either 0 (absent RB1 expression with RB1 expression present in normal cells serving as internal control), 1 (RB1 present), 2 (subclonal loss of RB1 expression), or 3 (cytoplasmic staining) or uninterpretable, which was scored as either 8 (RB1 absent but lacking adjacent internal control) or 9 (sample drop out). Representative images of RB1 expression patterns in tumor tissue are shown in Supplementary Fig. S2. Scoring was conducted by two pathologists (MK and EYK). Using two test TMAs with 192 cores, the interobserver agreement was 89.9% (κ = 0.816), including the assessment of whether the core was interpretable. When considering only the 156 cores that both pathologists deemed interpretable, the interobserver agreement was 98.1% (κ = 0.92).

### Molecular analyses

Subsets of patients with HGSC had additional molecular or immune data available (Supplementary Fig. S1), including tumor p53 protein expression status previously classified (38) as normal (wild-type) or abnormal (overexpression, complete absence, and cytoplasmic), germline *BRCA1* and *BRCA2* pathogenic variant status obtained from OTTA, *RB1* mRNA tumor expression and transcriptional subtypes of tumors using NanoString (35, 39), and CD8^+^ tumor-infiltrating lymphocyte (TIL) density was previously classified (40) based on the number of CD8^+^ TILs per high-powered field: negative (no TILs), low (<3 TILs), moderate (3–19 TILs), or high (≥20 TILs).

The MOCOG whole-genome and transcriptome sequencing dataset of 126 short-, moderate-, and long-term survivors was uniformly processed as previously described (27) and included detailed characterization of each tumor sample for inactivating alterations in *RB1* and HR pathway genes, including germline and/or somatic genetic alterations in *BRCA1*, *BRCA2*, *BRIP1*, *PALB2*, *RAD51C*, and *RAD51D* or promoter methylation of *BRCA1* and *RAD51C.* HRD status was assessed using the Classifier of Homologous Recombination Deficiency (CHORD) method (41), which uses specific base substitution, indel, and structural rearrangement signatures detected in tumor genomes to generate *BRCA1*-type and *BRCA2*-type HRD scores. Primary tumors were classified as either *BRCA1*-HRD & *RB1* altered, *BRCA1*-HRD & *RB1* wild-type, *BRCA2*-HRD & *RB1* altered, *BRCA2*-HRD & *RB1* wild-type, homologous recombination proficient (HRP) & *RB1* altered, or HRP & *RB1* wild-type.

### RNA-sequencing normalization and batch correction

Primary high-grade serous tubo-ovarian carcinoma (HGSC) samples were grouped according to *RB1* alterations and HRD status, as assessed previously using whole-genome sequencing (27) and the CHORD method (ref. 41; Supplementary Table S2). Matched RNA sequencing data were previously processed into gene expression counts as part of the prior MOCOG study (27). Briefly, raw count data were filtered to include only protein-coding genes. Lowly expressed genes were removed by converting the data to CPM (counts per million = number of reads mapped to a gene × 10^6^/total number of mapped reads), and only genes in which at least 10 samples had a CPM of greater than 0.5 were kept for further processing. The data were normalized using the trimmed mean of M values (TMM) method in edgeR (RRID:SCR_012802) and batch effects removed using the removeBatchEffect function of limma (RRID:SCR_010943). The batch correction was performed to remove batch effects while retaining group differences using limma’s removeBatchEffect function with the parameters [exp_data, batch = LibraryType, design = model.matrix(∼HR_RB1_status)], in which “exp_data” is the log_2_ TMM normalized data. The design of the study is shown in Supplementary Table S3.

### Differential gene expression analysis

Differentially expressed protein-coding genes were identified between sample groups of interest using DESeq2 (RRID: SCR_015687; ref. 42; v1.26.0), with batch effects accounted for in the model. In addition to characterizing the transcriptional profiles of tumors with *RB1* alterations and concomitant *BRCA1*- or *BRCA2*-type HRD relative to tumors with no alterations, DESeq2 was also used to evaluate alteration-specific transcriptional profiles by incorporating given alterations into the model to remove their signal (each comparison is shown in Supplementary Table S4). *HLA*-associated genes present in the differential expression results from DESeq2 were annotated to their relevant classes (43).

The R package fast gene set enrichment analysis (FGSEA v1.15.1; bioRxiv https://doi.org/10.1101/060012) was used to perform gene set enrichment analyses across comparison groups. Gene-level Benjamini–Hochberg adjusted *P* values obtained from DESeq2 were transformed to signed *P* values by converting them to a negative log_10_ value and applying the sign of the fold change. The signed *P* values were pre-sorted and fed into FGSEA via its function fgseaMultilevel (minSize = 15, maxSize = 500, gseaParam = 0, eps = 0) to generate enrichment scores and adjusted *P* values using the MSigDB (44) Hallmark gene sets (v7.4).

### Gene set variation analysis pathway enrichment

Gene lists for the cGAS-STING and Toll-like receptor signaling pathways were obtained from the PathCards database (45). Gene set enrichment was performed between the normalized batch corrected expression matrix and the pathways using the gene set variation analysis (GSVA) R package (v1.34.0) with parameters (method = “gsva,” kcdf = “Gaussian,” min.sz = 5, max.sz = 500).

### Cell culture

The AOCS patient-derived cell lines (AOCS1, AOCS3, AOCS7.2 AOCS9, AOCS11.2, AOCS14, AOCS16, AOCS22, and AOCS30) were established from ascites drained from patients with HGSC, as previously described (46). All AOCS cell lines were authenticated against matched patient germline DNA using short tandem repeat markers (STR, GenePrint10 System, Promega). Commercial cell lines OAW28 and CAOV3, categorized as likely HGSC (47), were purchased from the ATCC. Commercial lines were authenticated by comparing STR profiles (GenePrint10 System, Promega) with those published by online repositories [Cancer Cell Line Encyclopedia (48), The cBio Cancer Genomics Portal (49)] before use in experiments. Cell lines were confirmed to be free of *Mycoplasma* by PCR at each revival and after finishing experiments. Cell lines were maintained in a humidified incubator at 37°C and 5% CO_2_. All cell lines (aside from OAW28 and CAOV3) were cultured in RPMI 1640 (GIBCO, Carlsbad, CA) supplemented with 10% fetal bovine serum (FBS; Cytiva) and 1% penicillin–streptomycin–glutamine (GIBCO; Supplementary Table S5). OAW28 and CAOV3 were cultured in DMEM (GIBCO) supplemented with 10% FBS and 1% penicillin–streptomycin–glutamine, with the addition of 1 mmol/L sodium pyruvate and 20IU/l insulin for OAW28.

### Molecular characterization of cell lines

Complete cell line characterization details can be found in Supplementary Tables S5 and S6. The alteration status of genes of interest in AOCS cell lines was determined by either whole-genome (27) or targeted sequencing (7, 50) using established pipelines, and in commercial cell lines from published data (47) or The Cancer Cell Line Encyclopedia in cBioPortal (49, 51, 52). *BRCA* and *TP53* variants were classified as pathogenic if they were truncating (nonsense, splice site, or frameshift) alterations resulting in early stop codons or missense variants previously reported as pathogenic in ClinVar (53) or The TP53 Database (R20, July 2019, https://tp53.isb-cgc.org). *CCNE1* copy number in AOCS cell lines was analyzed by qPCR in triplicate on LightCycler 480 (Roche) using SYBR Green PCR mix (Applied Biosystems) as described previously (54). The expression status of RB1 and p16 was evaluated by Western blot (as below) and/or IHC. For IHC, FFPE cell line plugs were established by fixing approximately 6 × 10^7^ cells in 10% neutral buffered formalin overnight, transferring them into an agarose gel plug, and embedding them in paraffin. Duplicate cores were taken from each cell line plug and assembled in a paraffin block in the fashion of a tissue microarray. Cell line microarrays were sectioned, stained with antibodies (RB1, BD Pharmingen, BD Biosciences, clone G3-245; p16, Roche Ventana, CINtec, clone E6H4) and scored blinded by a pathologist. RB1 was classified as either absent, present, or uninterpretable; p16 was interpreted according to a three-tier scoring system as normal patchy, abnormal absent, or abnormal overexpressed.

### CRISPR-mediated gene knockout

*RB1* was inactivated using CRISPR-Cas9 (55) in cell lines with a pre-existing *BRCA1* alteration (AOCS7.2 and AOCS16) and a *BRCA1/2* wild-type cell line (AOCS1). Briefly, lentiviral transduction was performed using the FgH1t vector co-expressing Cas9, mCherry, and GFP and a doxycycline-inducible synthetic guide RNA (sgRNA) targeting *RB1* exon 7 or exon 8 (Supplementary Table S7). After sorting for double-positive cells (mCherry and GFP) by flow cytometry, expression of the sgRNA was induced with doxycycline (0.1 μg/mL media, Sigma-Aldrich, D3072) for 96 hours, and single cells were sorted into 96-well plates. Clones were expanded, and *RB1* status was confirmed by reduced/absent RB1 expression (Western blot, RT-qPCR) and Sanger sequencing of the targeted *RB1* exon. For control lines, *RB1* wild-type single-cell colonies without a CRISPR edit were used, as well as heterogeneous cell populations with transduced Cas9 and sgRNA of a scrambled DNA sequence (ref. 56; Supplementary Table S7).

Dual gene knockout of *RB1* and *BRCA1* was performed in AOCS30 using nucleofection (57–59) rather than lentivirus transduction. *BRCA1*, *RB1*, and control sgRNA sequences (CRISPRevolution sgRNA EZ Kit, Synthego) were designed as previously described (60, 61). Cells (5 × 10^5^) were trypsinized, washed twice with PBS, and incubated with the RNP complex (Alt-R S.p. Cas9 Nuclease purified Cas9 protein, Integrated DNA Technologies) for 10 min. Cell pellets were suspended with Nucleofector SE solution (Lonza Bioscience) and mixed with prepared Cas9/sgRNA RNP complex, which were transferred into the Nucleocuvette vessels (Lonza Bioscience). Nucleofection was conducted with the CL-120 Program in the 4D-Nucleofector X unit (Lonza Bioscience). Prewarmed medium was added to cells and incubated for 10 min in a humidified 37°C incubator with 5% CO_2_. Cells were transferred into six-well plates and cultured. Each cell line (AOCS30 NT, AOCS30 *BRCA1*KO, AOCS30 *RB1*KO, and AOCS30 *RB1BRCA1*KO) was passaged two times to expand following nucleofection, passed through a cell strainer (Falcon 40 μm) and plated at a low density (approximately 400 cells per 10-cm dish). After ∼14 days, independent colonies were trypsinized with cloning discs (Sigma-Aldrich) and expanded. Knockout efficiency was tested via qPCR as described below.

### Western blot analysis

Cells were washed with cold PBS and lysed in 1% SDS protein lysis buffer, with the addition of proteinase inhibitor and PhosSTOP solution (Roche) for phosphorylated protein. Protein concentrations were measured using Bio-Rad DC (detergent compatible) protein assay and 40-μg protein in SDS sample buffer and 2-mercaptoethanol was applied to Mini-PROTEAN TGX Gels 4% to 20% (Bio-Rad, Hercules, CA), subjected to gel electrophoresis at 115 V for 1 hour and 150 V for 10 minutes, transferred and blotted to polyvinylidene difluoride membranes for 10 minutes at 25 V with Trans-Blot Turbo Transfer System (Bio-Rad). Membranes were blocked with Odyssey Blocking Buffer (TBS; LI-COR Bioscience) for 1 hour at room temperature and incubated with the primary antibody (1:500–1:1,000 in TBS-T; Supplementary Table S8) overnight at 4°C. After washing the membranes for 3 × 10 minutes, they were incubated with the secondary goat anti-mouse or goat anti-rabbit AB coupled IR dye 680 RD or 800 CW (LI-COR, 1:10,000) for 1 hour and, after another three washing steps, membranes were imaged using the Odyssey Imaging System (LI-COR).

### RNA extraction and qPCR

Total RNA was extracted from cells using RNeasy Kits (QIAGEN) with on-column DNase digestion, of which 1 μg was reverse transcribed into cDNA using the SensiFAST cDNA Synthesis Kit (Meridian Bioscience). Transcript abundance was measured by real-time quantitative PCR (qPCR) using the SYBR Green qPCR assay (Applied Biosystems) on the LightCycler 480 (Roche), with each PCR performed in triplicate. Primer sequences are listed in Supplementary Table S9. Gene expression was estimated using the comparative threshold cycle method (ref. 62; ΔΔCt) against the average Ct value obtained for two control genes (*GAPDH* and *HPRT*).

### Cell viability assay

Cells were seeded at a density of 1 to 8 × 10^3^ per well, depending on growth rates, in 384-well microtiter plates (Corning) and incubated overnight. Cisplatin (100 μmol/L; Selleck Chemicals) and olaparib (80 μmol/L, Selleck Chemicals) were diluted in 3-fold steps to create a 10-point dose curve; paclitaxel (0.3 μmol/L, Selleck Chemicals) was diluted in 4-fold steps to create a 12-point dose curve. Following 72 h (cisplatin and paclitaxel) or 120 hour incubation (olaparib), cells were fixed in 2% paraformaldehyde for 10 minutes, washed with PBS, and stained with 0.19% Triton X solution containing DAPI (1:1,000; Sigma-Aldrich). Cell dispensing, media changes, and fixing and staining of cells were conducted robotically (BioTek Instruments, Winooski, VT). Drug dispensing was performed with ALH3000 Liquid Handler (PerkinElmer, Waltham, MA). To assess cell viability, the whole area of each well was captured at 10× magnification using a CX7-LZR instrument (Thermo Fisher Scientific), and images were analyzed using the CellProfiler v3.0 pipeline (RRID:SCR_007358). Low-quality out-of-focus images (4% of total images) were excluded by manual review before downstream analysis. Nonlinear regression drug curves were calculated using GraphPad Prism version 9.3.1 (RRID:SCR_002798), and differences in IC_50_ values were statistically measured by applying Akaike information criterion. Curve fit was compared between *RB1* WT and *RB1* KO clones by an extra sum-of-squares F test.

### Clonogenic survival assay

Cells (0.8 to 3 × 10^3^) were seeded in six-well plates (Corning) depending on cell doubling rates. After 12 hours, duplicate wells were treated with cisplatin, paclitaxel, or a combination of both drugs at the respective IC_50_ drug concentration, as determined by the 72-hour viability assay. Cells treated with media alone and with DMF solvent-containing media served as controls. After 16 days, cells were rinsed with PBS, fixed, and stained with 0.1% crystal violet and methanol for 20 min. The whole area of wells was captured in a brightfield at 2× magnification using the CX7 (Thermo Fisher Scientific), and the number of clones was assessed using the CellProfiler v3.0 software.

### Cell proliferation rates

Cells were counted using the Countess 3 Automated Cell Counter (Thermo Fisher Scientific) and seeded in 200-μL media in 96-well Corning plates in triplicate wells and incubated at 37°C. Cells were plated at three different densities (AOCS1 6 × 10^3^ to 8 × 10^3^ cells/well; AOCS7.2 8 to 12 × 10^3^ cells/well; AOCS16 14 to 18 × 10^3^ cells/well) according to a previously observed 20% cell confluency per well on day 1, and media changed after 5 days. The whole well area was captured in brightfield every 12 h for 9 days using real-live cell imaging (Incucyte Zoom) and cell proliferation rates were determined with Incucyte software. Growth rates were analyzed separately in triplicate wells with a starting confluency of between 15% and 25% in three independent experiments.

### Cell-cycle profiling

Cells were seeded in 12-well Corning plates at between 8 to 12 × 10^4^ cells/well (AOCS1 8 × 10^4^, AOCS7.2 10 × 10^4^, and AOCS16 12 × 10^4^ cells). After 24 h, each cell line was treated at half the concentration of the respective IC_50_ (determined in the above-described cell viability assay) of either cisplatin (AOCS1: 0.25 μmol/L; AOCS7.2: 0.25 μmol/L; AOCS16: 0.15 μmol/L), paclitaxel (AOCS1: 1.25 nmol/L; AOCS7.2: 50 nmol/L; AOCS16: 0.4 nmol/L) or a combination of both drugs for 24 h. Cells were rinsed with PBS, trypsinized to form a single-cell suspension, and fixed by adding ice-cold 70% ethanol drop-wise. Cells were pelleted and resuspended in a solution containing propidium iodide (0.05 mg/mL) and ribonuclease A (RNase A, Thermo Fisher EN0531, 10 mg/mL). Following 30–60 min of incubation at room temperature, DNA content was measured using the FACSCanto LSR II flow cytometer. Flowlogic software (Inivai) was used to analyze cell cycle distribution in the FL3-A channel by applying the Watson pragmatic algorithm (63).

### Statistical analyses

Cox proportional hazard models were used to estimate HRs with 95% confidence intervals (CI) using the “coxph” function of the R package *survival* (v3.2-7). Final models were fitted using Cox regression adjusted for age at diagnosis and Federation Internationale des Gynaecologistes et Obstetristes (FIGO) stage. A spline function was used for age at diagnosis with degree of freedom (*df*) 5 to account for the nonlinear effect of the continuous variable. Regression models were fitted separately by histotype. The HGSC regression models were also stratified by site of participant recruitment, and sites with fewer than 10 events within the study period were excluded. The endometrioid ovarian carcinoma (ENOC) regression model was not stratified by site due to the limited number of overall patients per site. We also ran sub-analyses adjusting for the extent of residual disease and ENOC grade. The OTTA survival dataset was right censored at 10 years from diagnosis to reduce the number of non-ovarian cancer–related deaths. In the final Cox regression model, there was evidence for deviation from the proportional hazard assumption, but the degree of deviation was not substantial when considered alongside the large sample size and Schoenfeld residuals. The Kaplan–Meier method was used to estimate and plot progression-free and overall survival probabilities, and the log-rank (Mantel–Cox) test was used to compare the survival duration between subgroups. In the Kaplan–Meier curves, the number of patients at risk on the date of diagnosis (time = 0) may be fewer than subsequent time intervals, owing to left truncation of follow-up resulting from delayed study enrollment at some OTTA sites. Differences in proportions of categorical features were assessed by either the *χ*^2^ or Fisher’s exact test as indicated. Differences in continuous variables were assessed by either a Wilcoxon rank-sum test or a Kruskal–Wallis test. All *in vitro* assays were performed across at least three independent experiments, and data are expressed as mean ± SEM as indicated, from a minimum of three independent measurements. All statistical tests were two-sided and considered significant when *P* < 0.05. Statistical analyses were performed using either GraphPad Prism (v9.3.1) or R (v3.6.3).

### Data availability

Genomic variants characterized in the MOCOG study (27), which also includes individuals from the International Cancer Genome Consortium Ovarian Cancer project (3), are available without access restrictions in Synapse under accession code syn34616347 (https://www.synapse.org/#!Synapse:syn34616347). The processed expression and methylation data from the MOCOG study are available without access restrictions in the Gene Expression Omnibus (https://www.ncbi.nlm.nih.gov/geo/) under accession code GSE211687. Unprocessed methylation data are available from Gene Expression Omnibus under the accession codes GSE65821 and GSE211687, with no access restrictions. DNA and RNA sequence data generated in the MOCOG and International Cancer Genome Consortium studies are available from the European Genome-phenome Archive (EGA) repository (https://ega-archive.org) under accession codes EGAS00001005984 and EGAD00001000877, subject to Data Access Committee approvals. Individual participant data from the OTTA study are not publicly available in keeping with the limitations imposed by patient consent and data privacy laws. All other data are provided within the supplementary data files or available upon request to the corresponding author.

## Results

### Loss of RB1 expression is most frequent in HGSC

**Figure 1.**
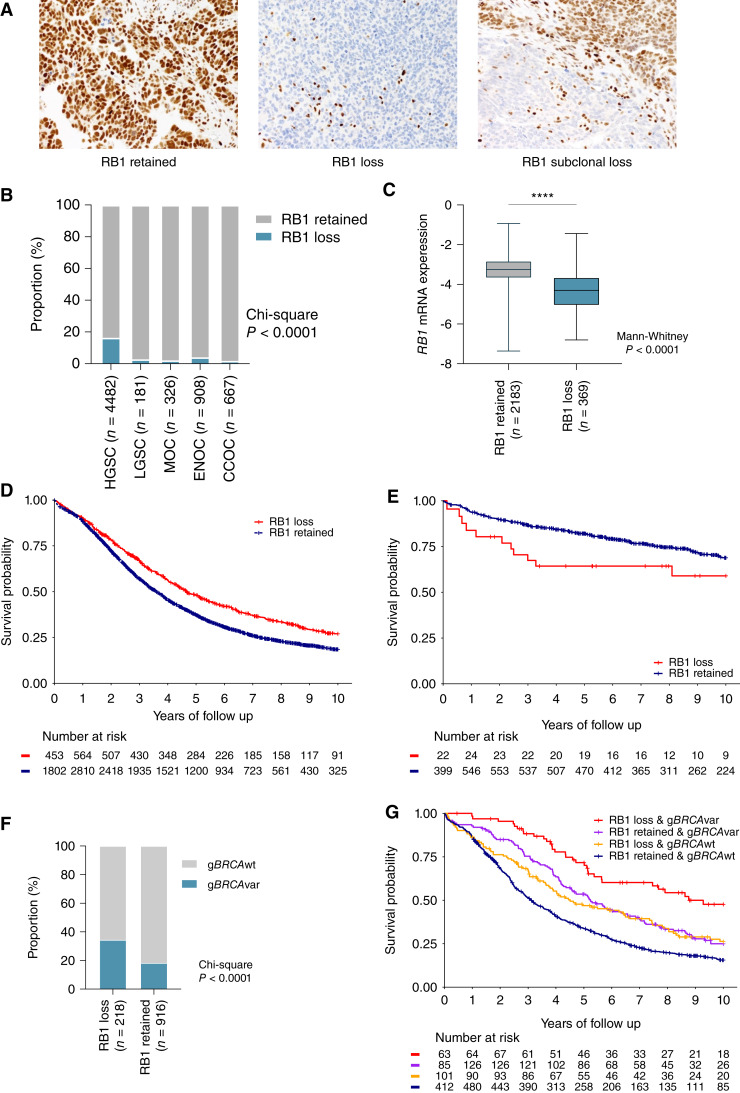
Expression of RB1 and survival associations across ovarian cancer histotypes. **A,** Representative images of IHC detection of RB1 expression in ovarian carcinoma tissues, showing examples of the three most common expression patterns: retained, lost, and subclonal loss. **B,** Proportion of patients with loss or retention of RB1 protein expression in tumor samples by ovarian cancer histotypes. *χ*^2^*P* value reported for difference in proportions across all histotypes. CCOC, clear cell ovarian cancer; LGSC, low-grade serous carcinoma; MOC, mucinous ovarian cancer. **C,** Boxplots show *RB1* mRNA expression (NanoString) by RB1 protein expression status; lines indicate median and whiskers show range (Mann–Whitney test *P* value reported). Kaplan–Meier analysis of OS in patients diagnosed with HGSC (**D**) and ENOC (**E**) stratified by tumor RB1 expression. **F,** Frequency of germline *BRCA* wild-type (g*BRCA*wt) and germline *BRCA* pathogenic variants (g*BRCA*var) in patients with HGSC stratified by RB1 protein expression. χ^2^*P* value is reported. **G,** Kaplan–Meier estimates of overall survival in patients with HGSC by combined germline *BRCA* and tumor RB1 expression status.

RB1 protein expression was assessed by IHC in tumor samples from 7,436 patients with ovarian carcinoma using TMAs from 20 centers participating in the OTTA consortium (Table 1; Supplementary Tables S1 and S10). RB1 tumor expression was classified as either retained or lost in 6,564 samples, with 872 samples excluded that had either subclonal loss (*n* = 66), cytoplasmic (*n* = 17), or uninterpretable results (*n* = 789) due to either sample drop out or the absence of an internal positive control (Fig. 1A).

RB1 loss was most frequent in HGSC (16.4%), followed by endometrioid ovarian carcinoma (ENOC; 4.1%, *χ*^2^*P* < 0.0001; Fig. 1B). Loss of RB1 expression was less frequent in all other histotypes (1.8%–2.8%). *RB1* mRNA expression was also assessed by NanoString in a subset of HGSC tumors (*n* = 2,552) and was significantly associated with RB1 protein expression (Fig. 1C, *P* < 0.0001).

### RB1 loss is associated with longer survival in HGSC

Loss of RB1 protein expression was associated with longer OS in patients with HGSC (HR, 0.74; 95% CI, 0.66–0.83; *P* = 6.8 × 10^−7^; Table 2) following multivariate analysis adjusting for stage and age at diagnosis and stratified by study. The effect size was similar after adjustment for the extent of residual disease following cytoreduction (HR, 0.66; 95% CI, 0.55–0.78; *P* = 1.1 × 10^−6^; Supplementary Table S11). Patients with HGSC were comparable in terms of stage regardless of RB1 loss or retained expression (*P* = 0.9246); however, those with RB1 loss had a younger age at diagnosis (median 59 vs. 61 years, *P* = 0.0003; Supplementary Table S12). The median OS was 4.7 years for patients with RB1 loss compared with 3.6 years for those with retained RB1 expression (Fig. 1D).

**Table 2.** Multivariate analysis of molecular alterations and OS in patients with HGSC and ENOC.

Histotype	Feature	Category	No. patients	(events, %)	HR	(95% CI)	*P*	*P*_*int*_
HGSC^a^^,^^b^								
	RB1							
		Retained	3,453	(71.3)	1	[Reference]		
		Loss	686	(61.1)	0.74	(0.66–0.83)	6.8 × 10^−7^	
	RB1 and *BRCA* status							
		RB1 retained & g*BRCA*wt	714	(76.3)	1	[Reference]		0.24
		RB1 loss & g*BRCA*wt	135	(60.7)	0.74	(0.57–0.96)	0.023	
		RB1 retained & g*BRCA*var	159	(67.9)	0.69	(0.55–0.86)	0.001	
		RB1 loss & g*BRCA*var	70	(42.9)	0.38	(0.25–0.58)	5.2 × 10^−6^	
ENOC^a^								
	RB1							
		Retained	649	(22.7)	1	[Reference]		
		Loss	28	(39.3)	2.17	(1.17–4.03)	0.014	
	RB1 and p53							
		RB1 retained & p53 normal	492	(17.5)	1	[Reference]		0.698
		RB1 retained & p53 abnormal	58	(36.2)	2.26	(1.38–3.71)	0.001	
		RB1 loss & p53 normal	11	(27.3)	1.77	(0.56–5.65)	0.332	
		RB1 loss & p53 abnormal	12	(58.3)	5.34	(2.43–11.8)	<0.001	

Abbreviation: *P*_int_, *P* for interaction.

a Adjusted for stage and age at diagnosis.

b Stratified by study.

In contrast to HGSC, loss of RB1 expression in tumors from patients with ENOC was associated with advanced stage (*P* = 0.0003), high-grade (*P* < 0.0001), and poorer survival (HR, 2.17, 95% CI, 1.17–4.03, *P* = 0.0140; Table 2; Fig. 1E; Supplementary Table S13). RB1 loss and abnormal p53 protein expression, which is highly predictive of *TP53* mutation (64), were strongly correlated (χ^2^*P* < 0.0001; Supplementary Fig. S3A). *TP53* mutation is known to be associated with inferior survival in patients with ENOC (38, 65); however, we note that combined RB1 loss and abnormal p53 expression were associated with the shortest patient survival (median OS 3.0 years; Supplementary Fig. S3B). Although high-grade ENOC showed a higher proportion of RB1 loss (Supplementary Table S13), RB1 loss alone was not significantly associated with survival after adjusting for grade (*P* = 0.133) or the extent of residual disease (*P* = 0.107; Supplementary Tables S11 and S14). Nevertheless, the subset of patients with RB1 loss and p53 abnormal ENOC had the poorest survival, regardless of grade (HR, 4.91; 95% CI, 1.95–12.4; *P* < 0.001) and residual disease (HR, 3.78; 95% CI, 1.12–12.64; *P* = 0.031; Supplementary Tables S11 and S14).

### Combined RB1 loss and germline *BRCA* deficiency is associated with exceptionally good survival

We previously observed that the co-occurrence of somatic RB1 protein loss and *BRCA1* or *BRCA2* alteration (somatic or germline) was associated with longer progression-free survival (PFS) and OS in HGSC (7). Here, germline *BRCA1* and *BRCA2* status was available for 1,134 patients with HGSC for which we had RB1 IHC data (Supplementary Fig. S1). Consistent with having a younger age of diagnosis, patients with RB1 loss were more likely to have concurrent g*BRCA*var than those with retained RB1 expression (Fig. 1F, *χ*^2^*P* < 0.0001; Supplementary Fig. S3C). Patients with both RB1 loss and g*BRCA*var had a 62% reduced risk of death compared with those with g*BRCA*wt and retained RB1 (HR, 0.38; 95% CI, 0.25–0.58; *P* = 5.2 × 10^−6^; Table 2). This association remained significant after adjustment for surgical outcome (*P* < 0.001; Supplementary Table S11). The median OS of g*BRCA*var with RB1 loss was three times longer than g*BRCA*wt with RB1 retained tumors (median OS 9.3 vs. 3.1 years, respectively), whereas the median OS was 5.2 years for g*BRCA*var with retained RB1 expression and 4.5 years for g*BRCA*wt with RB1 loss (Fig. 1G; Supplementary Table S15). Although there were too few patients to differentiate between *BRCA1* and *BRCA2* variants in the primary regression analysis, a stronger association between RB1 loss and survival was seen in patients with a g*BRCA1*var (median OS 9.3 years RB1 loss vs. 4.7 years RB1 retained) compared with those with a g*BRCA2*var (median OS 8.6 years RB1 loss vs. 5.8 years RB1 retained; Supplementary Fig. S3D; Supplementary Table S16).

### Enhanced response to chemotherapy in cells with impaired *BRCA* and *RB1* function

To investigate whether co-occurrence of *RB1* and *BRCA* alterations enhances sensitivity to standard-of-care ovarian cancer drugs, nine patient-derived HGSC cell lines with confirmed pathogenic *TP53* mutation and known *RB1* and *BRCA* status were treated with cisplatin, paclitaxel, and olaparib (Supplementary Fig. S4A and S4B; Supplementary Table S17). AOCS14, the only cell line with a g*BRCA1*var and concomitant loss of RB1 expression, showed the best response to cisplatin and olaparib and was the second most sensitive cell line to paclitaxel. In contrast, AOCS11.2, a line with *BRCA1* promoter methylation and loss of RB1 expression, was relatively resistant to paclitaxel and olaparib. Among cell lines with intact RB1 protein expression and *BRCA* wild-type background, AOCS3 was resistant to cisplatin, paclitaxel, and olaparib.

**Figure 2.**
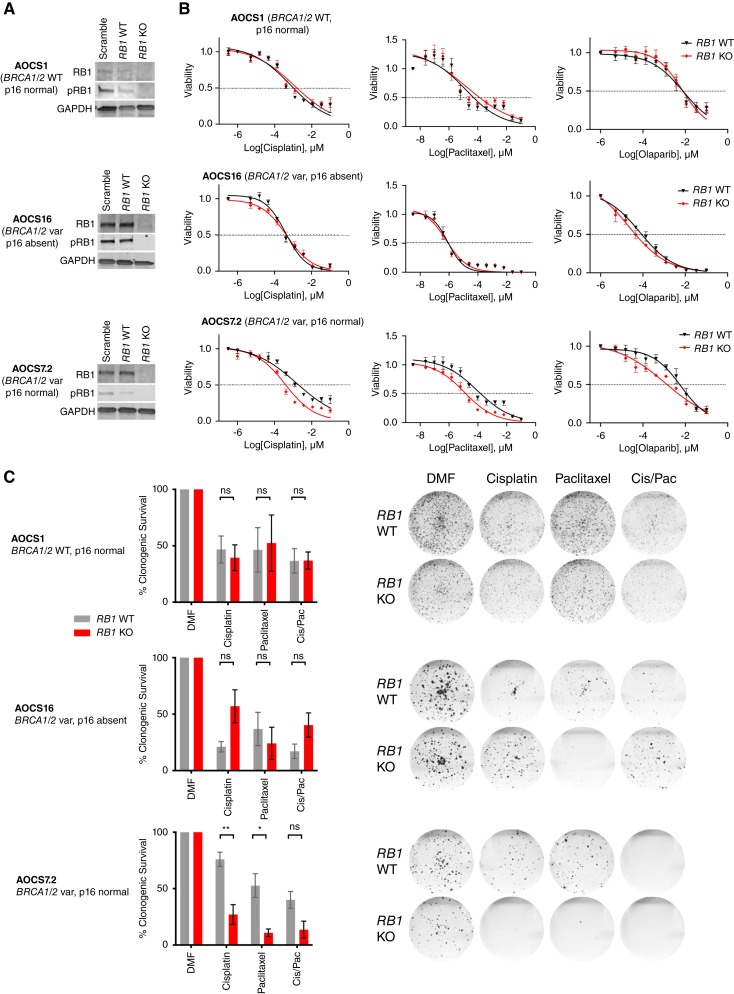
Sensitivity to therapeutic agents in *BRCA1*-altered cell lines with *RB1* knockout. **A,***RB1* was knocked out using CRISPR/Cas9 in three patient-derived Australian Ovarian Cancer Study (AOCS) HGSC cell lines with either wild-type or altered *BRCA1* (*BRCA1* var) background. Representative Western Blots show protein levels of RB1 and phosphorylated RB1 (pRB1) compared with GAPDH loading control in single-cell cloned, homozygous *RB1* wild-type (WT) and knockout (KO) colonies in comparison with heterogeneous populations with a scramble single guide RNA (sgRNA). Independent blots were used for RB1 and pRB1. **B,** Cell viability was compared between *RB1* WT and KO clones following treatment with cisplatin (72 h), paclitaxel (72 h), or olaparib (120 h). Nonlinear regression drug curves are shown; *P* values are shown in Supplementary Table S18 (*n* = 3). Error bars indicate ± SEM; for some values, error bars are shorter than the symbols and thus are not visible. **C,** Proportion of surviving colonies following 16 days of treatment with cisplatin, paclitaxel, or a combination of both (Cis/Pac; with half of the IC50 determined per drug and cell line respectively) relative to DMF vehicle control (*n* = 3 replicates). Data are presented as mean ± SEM. Mean values were compared by Student’s *t* test (ns, not significant; *, *P* < 0.05; **, *P* < 0.01). Representative scans of the fixed cell colonies stained with crystal violet are shown for each condition.

Except for the chemo-naïve cell lines AOCS30 and AOCS14, all other lines were derived from patients previously treated with chemotherapy. As the evaluation of HGSC cell lines with existing *RB1* alterations may have been confounded by their prior, differential exposure to chemotherapy we therefore characterized responses in isogenically matched lines deleted of *RB1* and/or *BRCA1.* We first inactivated *RB1* in two *BRCA1*-altered (AOCS7.2, AOCS16) and one wild-type line (AOCS1) using CRISPR-Cas9 (Fig. 2A; Supplementary Fig. S5A). *RB1*-knockout clones of the *BRCA1*-altered cell line AOCS7.2 had enhanced sensitivity to cisplatin and paclitaxel compared with *RB1* wild-type clones, which was observed both in short-term drug assays (72 h; Fig. 2B) and longer-term clonogenic survival assays (12 days; Fig. 2C). In this cell line, sensitivity to cisplatin, paclitaxel, and olaparib was increased after *RB1* knockout (cisplatin IC_50_ 1.56 vs. 0.38 μmol/L, *P* = 0.01; paclitaxel IC_50_ 92.0 vs. 11.8 nmol/L, *P* = 0.0004; olaparib IC_50_ 6.1 vs. 1.1 nmol/L, *P* = 0.0005; Supplementary Table S18). Furthermore, significantly fewer colonies grew in this *BRCA1*-altered cell line after *RB1* knockout upon treatment with cisplatin (*P* = 0.01), paclitaxel (*P* = 0.02), or a combination of both drugs (*P* = 0.067) in a clonogenic survival assay (*n* = 3). This effect was not apparent in the *BRCA* wild-type line (AOCS1) or the other *BRCA1*-altered line (AOCS16), except for an increase in sensitivity to olaparib seen in AOCS16 upon RB1 depletion (olaparib IC_50_ 0.072 vs. 0.022 nmol/L, *P* = 0.04; Supplementary Table S18). Western blot and IHC analysis (Supplementary Fig. S5A) found that AOCS16 lacked expression of p16, which may functionally disrupt the RB1 pathway irrespective of an *RB1* knockout (66).

Given that RB1 plays a central role in the negative control of the cell cycle (66, 67), we tested whether the enhanced chemosensitivity of *RB1* knockout AOCS 7.2 cells was associated with increased cell division. Live cell imaging showed similar growth rates of *RB1* wild-type and knockout clones of all three isogenically matched HGSC cell lines (Supplementary Fig. S5B). In both *BRCA* wild-type and *BRCA1*-altered cell lines, *RB1* knockout did not alter cell cycle distribution at baseline or after 24 hours of cisplatin treatment (Supplementary Fig. S5C). Paclitaxel treatment resulted in a larger proportion of cells with a tetraploid DNA content in *RB1* knockout cells compared with *RB1* wild-type cells, indicating arrest in the G_2_ or M phase of the cell cycle. This effect was observed in all cell lines independent of *BRCA* or p16 status; however, the arrest was more profound in the AOCS7.2 cell line (AOCS1, G_2_/M difference 8.59% ± 4.73%, *P* = 0.144; AOCS16, G_2_/M difference 8.13% ± 4.45%, *P* = 0.142; AOCS7.2: G_2_/M difference 14.49% ± 3.99%, *P* = 0.022; Supplementary Fig. S5C).

We extended our analysis of isogenically matched pairs by inactivating *BRCA1* and/or *RB1* in the chemo-naïve cell line AOCS30. Although we were readily able to establish *RB1* knockout lines, all *BRCA1* targeted clones were hemizygous for *BRCA1* deletion and retained *BRCA1* expression (Supplementary Table S19), suggesting that engineered homozygous loss of *BRCA1* was cell lethal, even in a tumor type in which *BRCA1* loss is frequently observed (68).

### Genomic and transcriptional landscape of HGSC with combined inactivation of *BRCA* and *RB1*

**Figure 3.**
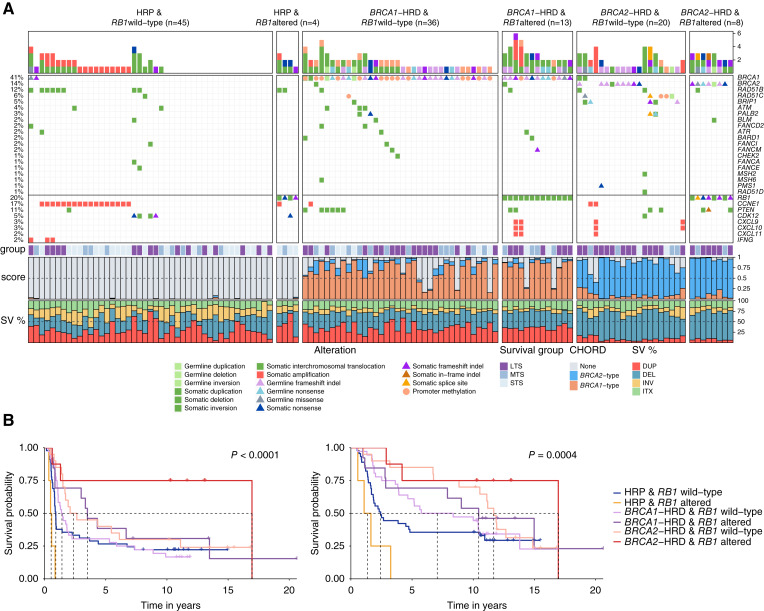
Genomic landscape of high-grade serous ovarian tumors with co-occurring *BRCA* and *RB1* alterations. **A,** Pathogenic germline and somatic alterations in HR and DNA repair genes detected by whole-genome sequencing and DNA methylation analysis of 126 primary HGSC samples (27) are shown, as well as alterations in immune genes and *CCNE1*. Samples are grouped by HR and *RB1* status. Bars at the top indicate the number of alterations in each listed gene per patient. Patients are annotated with survival group (LTS, long-term survivor, OS > 10 years; MTS, mid-term survivor, OS 2–10 years; STS, short-term survivor, OS < 2 years), tumor CHORD (41) scores, and the proportion of structural variant (SV) type (DEL, deletion; DUP, duplication; INV, inversion; ITX, intra-chromosomal translocation). **B,** Kaplan–Meier estimates of progression-free survival (left) and overall survival (right) of patients according to HR status (*BRCA1*-type HRD; *BRCA2*-type HRD; or HRP tumors) and *RB1* status (altered vs. wild-type).

To further understand how RB1 loss may impact the biology of HGSC with co-loss of *BRCA1* or *BRCA2*, we explored matched whole-genome and transcriptome data of primary HGSC tumors in the MOCOG cohort (27) of 126 short-term (OS < 2 years), moderate-term (OS ≥ 2 to <10 years), and long-term (OS ≥ 10 years) survivor patients (Supplementary Fig. S1). Each tumor genome was classified according to their HRD and *RB1* status, resulting in six groups: *BRCA1*-HRD & *RB1* altered (*n* = 13); *BRCA1*-HRD & *RB1* wild-type (*n* = 36); *BRCA2*-HRD & *RB1* altered (*n* = 8); *BRCA2*-HRD & *RB1* wild-type (*n* = 20); HRP & *RB1* altered (*n* = 4); or HRP & *RB1* wild-type (*n* = 45; Fig. 3A).

The cohort had been selected for a long-term survivor study (27) and hence was enriched for patients with very long survival. Among patients with *BRCA2*-HRD, those with *RB1* alterations had longer OS (median OS 17.0 years) compared with those without *RB1* alterations (median OS 11.7 years, *P* = 0.0004; Fig. 3B). Similarly, patients with *BRCA1*-HRD and *RB1* alterations survived longer (median OS 10.4 years) than those with an intact *RB1* gene (median OS 7.1 years). There were few HRP tumors with *RB1* alterations; however, these patients had a worse survival (median OS 1.4 years) compared with the HRP group with no *RB1* alteration (median OS 2.4 years).

Examination of genomic features revealed relatively similar patterns within *BRCA1*-HRD and *BRCA2*-HRD groups, although there were a few discriminatory features identified between those with and without *RB1* alterations (Supplementary Figs. S6 and S7; Supplementary Table S2). For example, the *BRCA1*-associated rearrangement signature Ovary_G (69) was more enriched in *BRCA1*-HRD tumors with *RB1* alterations compared with those without (*P* = 0.039). Among *BRCA2*-HRD tumors, the mutational signatures DBS6 (unknown etiology) and SBS3 (associated with HRD; ref. 70) were higher in *RB1*-altered tumors compared with non-altered tumors, although this was not significant (*P* = 0.082 and *P* = 0.1 respectively). Concordantly, the average *BRCA1*- and *BRCA2*-type CHORD scores (41) were highest in *BRCA1*- and *BRCA2*-HRD tumors with *RB1* alterations respectively, indicating a higher probability of HRD. As described previously (71), *CCNE1* gene amplifications were absent in tumors with both HRD and *RB1* alterations (*P* = 0.0006; Supplementary Fig. S8).

**Figure 4.**
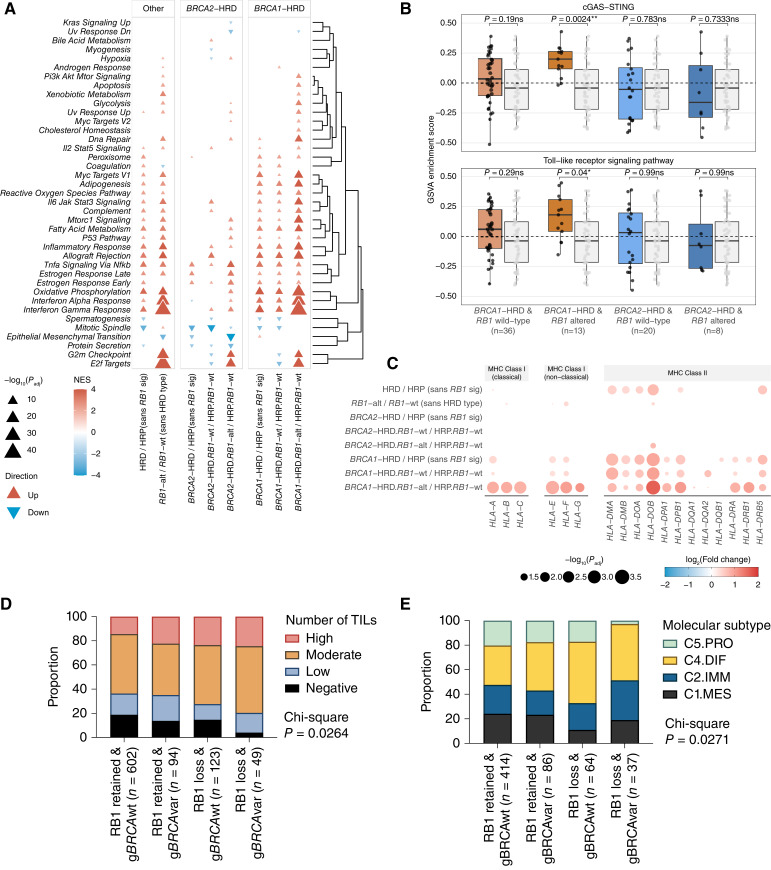
Characterization of HGSC with co-loss of RB1 and *BRCA*. **A,** GSEA indicating up- and downregulated pathways in tumors according to *BRCA* and *RB1* status. *RB1*-alt, *RB1* altered; *RB1*-wt, *RB1* wild-type. **B,** Boxplots comparing GSVA pathway enrichment scores of the cGAS-STING and Toll-like receptor signaling pathways between molecular subgroups; points represent each sample, boxes show the interquartile range (25th–75th percentiles), central lines indicate the median, and whiskers show the smallest/largest values within 1.5 times the interquartile range. Colored boxes with black points indicate the HRD and/or *RB1* altered groups, whereas the gray boxes with gray points indicate the HRP and *RB1* wild-type group. *P* values were calculated using a two-sided Mann–Whitney-Wilcoxon test. Benjamini–Hochberg adjusted *P* values are shown above each pairwise comparison (*, *P* < 0.05; **, *P* < 0.01; ns, *P* ≥ 0.05). **C,** Bubble plot summary of *HLA* gene expression comparisons using DESeq2 between HGSC tumors grouped by HRD and/or *RB1* status as shown. The size of the bubbles corresponds to the negative log_10_ Benjamini–Hochberg adjusted *P* value (*P*_adj_) and only values with *P*_adj_ ≤ 0.1 are shown. The color and intensity correspond to the log_2_fold change. Genes are grouped by their classes. **D,** Proportion of TILs in HGSC tumors grouped by RB1 protein expression and *BRCA* germline status. *χ*^2^*P* value is indicated. **E,** Proportion of tumors classified as each HGSC molecular subtype (13) grouped by RB1 expression and *BRCA* germline status. χ^2^*P* value is indicated. C4.DIF, C4/differentiated subtype; C2.IMM, C2/immunoreactive subtype; C1.MES, C1/mesenchymal subtype; C5.PRO, C5/proliferative subtype.

We hypothesized that tumors with combined HRD and *RB1* loss may have unique transcriptional profiles. To explore this, we compared gene expression profiles between each HRD/*RB1* group and the reference set of tumors that were HRP and *RB1* wild-type (Supplementary Table S4; Supplementary Fig. S9). There was significant enrichment of MSigDB hallmark gene sets among genes differentially expressed in *BRCA1*-HRD tumors with *RB1* alterations, the most prominent being IFNγ response (up), IFNα response (up), oxidative phosphorylation (up), and E2F targets (up; adjusted *P* < 0.0001; Fig. 4A). The differentially expressed genes identified between *BRCA2*-HRD/*RB1* altered tumors and the reference set were significantly enriched for the MSigDB hallmark gene sets: E2F targets (up), epithelial–mesenchymal transition (down), G_2_–M checkpoint (up), and TNFα signaling via NF-κB (up; adjusted *P* < 0.0001).

Inference of immune cell subsets (72) showed enrichment of follicular helper T cells in *BRCA2*-HRD/*RB1* altered tumors (adjusted *P* = 0.094), and regulatory T cells in *BRCA1*-HRD/*RB1* altered tumors (adjusted *P* = 0.016), compared with HRP/*RB1* wild-type tumors (Supplementary Fig. S10; Supplementary Table S20). Upregulation of immune-related transcription was particularly apparent in the *BRCA1*-HRD/*RB1* altered tumors, which were the only subgroup to show increased cGAS-STING (*P* = 0.0024) and Toll-like receptor signaling pathway activity (*P* = 0.04; Fig. 4B). Concordantly, *BRCA1*-HRD/*RB1*-altered tumors displayed evidence of increased expression of MHC Class I molecules (Fig. 4C).

As enhanced tumor cell proliferation has been associated with long-term survival in HGSC (7, 27), and loss of RB1 might accelerate proliferation (32), we evaluated the expression of proliferation markers across the *RB1* and *BRCA* subgroups. *BRCA1*-HRD tumors with *RB1* alterations had significantly higher mRNA levels of the cell proliferation-related genes *PCNA* (proliferating cell nuclear antigen) and *MCM3* (minichromosome maintenance complex component 3) compared with *BRCA1*-HRD tumors without *RB1* alterations (*P* < 0.0001; Supplementary Fig. S7). However, there were no significant differences in the proportion of Ki67-positive cancer cell nuclei (*P* = 0.3297) across the subgroups (Supplementary Fig. S7), which was previously quantified by immunohistochemistry (7) in a subset of primary tumors (*n* = 59).

### Patients with germline *BRCA* deficiency and somatic loss of RB1 tumor expression show elevated immune activity

Having observed that HGSC with combined RB1 loss and HRD have enrichment of transcriptional signatures associated with an enhanced immune response, we accessed existing IHC data (40) to determine the prevalence of CD8^+^ TILs in HGSC samples that also had RB1 protein expression and *BRCA* germline status (*n* = 868). Patients with g*BRCA*var and RB1 loss had a significantly higher proportion of tumors (79.6%) with moderate and high densities of CD8^+^ TILs, compared with g*BRCA*var with retained RB1 (64.9%), g*BRCA*wt with RB1 loss (72.4%), and g*BRCA*wt with retained RB1 (63.6%, *P* = 0.0264; Fig. 4D). Tumors with complete absence of CD8^+^ TILs were the least frequent in g*BRCA*var with RB1 loss (4.1%) compared with the other groups (13.8% of g*BRCA*var with retained RB1 tumor expression, 14.6% of g*BRCA*wt with RB1 tumor loss, and 18.8% of g*BRCA*wt with retained RB1 tumor expression).

Gene expression-based molecular subtypes (13, 39) also differed by RB1 and *BRCA* status (*P* = 0.0271, *n* = 601; Fig. 4E). As expected, there was enrichment for the C2/immunoreactive subtype, a subtype characterized by the presence of intratumoral CD8^+^ T cells and good survival, in g*BRCA*var with RB1 loss (32.4%) compared with the other subgroups (between 19.8% and 23.4%). Additionally, tumors with RB1 loss were enriched for the C4/differentiated molecular subtype, a subtype characterized by cytokine expression and good survival, regardless of *BRCA* status (45.9% in g*BRCA*var with RB1 loss, 50.0% in g*BRCA*wt with RB1 loss, 39.5% in g*BRCA*var with retained RB1, 32.1% of g*BRCA*wt with retained RB1). g*BRCA*var with RB1 loss also had the lowest proportion of the C5/proliferative molecular subtype (2.7% vs. 17.2%–20.3% in the other groups), a subtype associated with diminished immune cell infiltration and poor survival (13, 20).

## Discussion

Identifying the determinants of long-term patient survival, particularly in cancers with a generally unfavorable prognosis such as HGSC, may reveal novel therapeutic targets and inform personalized treatment strategies (8). Improved survival associated with RB1 loss has been described previously in HGSC (35, 36, 73), including in the context of co-occurring HR gene alterations (7, 74), but the underlying factors contributing to this survival benefit have not been studied to date. We assessed tumor samples from a cohort of more than 7,000 patients with ovarian carcinoma, including a subset with high-resolution genomic data, to understand how RB1 loss may impact therapeutic response and patient survival.

Alteration of the RB1 pathway is a frequent event in tumorigenesis, including loss of regulators such as p16, activation of D- and E-type cyclins and their associated cyclin-dependent kinases, and loss of RB1 itself (reviewed in ref. 75). Our study showed that RB1 loss is associated with longer survival in patients with advanced-stage HGSC, but by contrast, loss of RB1 in ENOC was associated with shorter survival, particularly in combination with p53 mutation, suggesting that loss of RB1 and *TP53* mutation have a compounding negative impact on survival in patients with ENOC. This casts doubt on the rationale of grouping p53 abnormal ENOC with HGSC in clinical trials. Despite suggestions from its endometrial counterpart (76), we are not aware of large studies confirming HRD in high-grade or p53 abnormal ENOC and the only rationale to combine them with HGSC may be a historical problem in the pathologic classification of these tumors (77). Similar to ENOC, in prostate cancer, RB1 loss is associated with poorer survival: early somatic co-deletion of *BRCA2* and *RB1* is associated with an aggressive, castration-resistant prostate cancer subtype characterized by epithelial-to-mesenchymal transition and shorter survival (30). RB1 loss seems to facilitate lineage plasticity and, with p53-commutation, leads to an androgen-independent prostate cancer phenotype (78, 79) and consequently resistance to anti-androgen therapy.

Triple-negative breast cancer (TNBC) provides an important parallel to the findings for RB1 loss in HGSC. In TNBC, RB1 loss is most common in the basal-like subtype, in which *BRCA1* inactivation is associated with frequent *RB1* gene disruption and RB1 loss (29). RB1 loss alone, as well as co-occurrence with *BRCA1* promoter hypermethylation, is associated with a favorable chemotherapy response and outcome (28, 80–82). Notably, TNBC and HGSC are more similar than the cancers that they are grouped with anatomically, sharing gene expression patterns, genetic drivers including *BRCA1* and *BRCA2*, ubiquitous loss of *TP53*, extensive copy number variation, and susceptibility to platinum-based chemotherapy (83, 84). Taken together, the relationship between RB1 loss and patient survival seems to be dependent on the histotype and/or the molecular context (85).

Some, but not all, TNBC and early metastatic prostate cancers are associated with germline variants in *BRCA1*, *BRCA2*, and other genes involved in HR DNA repair. However, previous tumor studies of RB1 expression have not also defined the HRD status of individual samples. A strength of this study was the known *BRCA* germline status of 1,134 of the patients with HGSC for which we also had RB1 protein expression, and this revealed the strong association of co-alteration in either *BRCA1* or *BRCA2* and *RB1* with survival, regardless of the extent of residual disease following primary debulking surgery. In addition to germline pathogenic variants in *BRCA1* or *BRCA2*, germline or somatic inactivation of other genes involved in HR DNA repair, such as *RAD51C*, can result in a similar molecular phenotype, characterized by distinct genomic scarring (27). Using whole-genome sequence data, we determined the likely tumor HRD status in a subset of 126 tumors using an algorithm that recognizes genomic scarring associated with HRD (Fig. 3A), rather than simply designating *BRCA* alteration status, which does not account for all mechanisms of HR repair inactivation (86). Although the number of samples with RB1 loss and HR proficiency was small, the very poor outcome we observed within this group suggests that RB1 loss may only be associated with better survival in an HRD background. Validation of this finding in a larger cohort may further inform how RB1 loss could favorably influence survival in certain histologic and molecular contexts.

We have previously noted that enhanced proliferation in HGSC is associated with long-term survival (7, 27), and it is reasonable to suggest that RB1 loss may be imparting an effect through deregulating the cell cycle. However, data on the effect of RB1 loss on proliferation in HGSC tumors and cancer cell lines are inconsistent. *RB1* knockout in our HGSC cell lines did not cause cell cycle alterations in the absence of treatment, and despite differences in proliferative markers at the mRNA level, there was no significant difference in the proportion of Ki67 positive nuclei between tumors with or without RB1 protein expression. In a recent OTTA study, Ki67 expression was not associated with survival in HGSC; however, there was a strong correlation between loss of RB1 and the proliferative marker MCM3 (87), which may provide a more accurate measure of tumor cell proliferation than Ki67 (88).

In addition to its role in driving progression through the G_1_ stage of the cell cycle, RB1 has non-canonical functions. RB1 has been shown to participate in HR DNA repair through interactions with BRG1 and ATM (34). A recent pan-cancer study (89) found that combined loss of *TP53* and *RB1* was associated with a particularly high genome-wide loss-of-heterozygosity score, one of the key elements of genomic scarring associated with HRD. In our whole-genome analysis, HGSC tumors with dual loss of HRD and *RB1* did not exhibit an overall higher mutation burden; however, we did observe elevated levels of mutational signatures associated with HRD, which may be evidence of compounding DNA repair defects. It remains possible that the combined inactivation of *RB1* and HR genes contributes to enhanced chemotherapy response and/or an impaired ability for tumor cells to develop therapy resistance.

When we evaluated a set of patient-derived HGSC lines, those with *BRCA1* and *RB1* alterations were most sensitive to cisplatin and olaparib. Knockout of *RB1* in the AOCS 7.2 cell line, which had a pre-existing *BRCA1* alteration, resulted in an increase in chemosensitivity, consistent with the notion that co-loss enhances chemotherapy response (7). Unfortunately, despite considerable efforts, we were unable to generate a larger series of isogenically matched cell lines with combinations of conditional knockouts of *RB1* and *BRCA1,* as all surviving clones retained at least one *BRCA1* allele. *BRCA1* loss is embryonic lethal and engineered loss in cell lines has been reported as lethal elsewhere, including in the human haploid cell line HAP1 (68).

The survival benefit associated with RB1 loss was more pronounced in patients with germline *BRCA1* variants compared with those with germline *BRCA2* variants. This is somewhat unexpected, given the increasing evidence that *BRCA2* loss seems to confer a greater survival advantage than *BRCA1* loss, especially at 10 years since diagnosis (24, 27). These differences could be partially explained by the increased immune activity observed in tumors with RB1 loss, particularly prevalent in *BRCA1*-HRD/*RB1*-altered HGSC. This group showed the strongest cGAS-STING pathway activity, suggesting that RB1 loss may further enhance cytosolic DNA-dependent type I IFN signaling, which is thought to be associated with *BRCA1* loss in HGSC (90). RB1 has been shown to inhibit innate IFNβ production in immunocompetent mice (91) and *RB1* deficiency triggered an increased IFNβ and IFNα secretion. Co-mutation of *RB1* and *TP53* was recently found to be associated with an enhanced response to the immune checkpoint inhibitor atezolizumab in metastatic urothelial bladder cancer (92). Similarly, a case report described a complete response to atezolizumab in heavily pre-treated, RB1-negative TNBC (93). This generates the hypothesis that RB1 loss could predict response to such therapies in HGSC, given that this tumor type ubiquitously harbors *TP53* mutations (94). However, a recent biomarker study in patients with ovarian cancer treated with atezolizumab or placebo and standard chemotherapy found that deleterious mutations in *RB1* were prognostic for a better PFS, regardless of the addition of atezolizumab (95). Although it seems RB1 loss alone may not be predictive of response to the PDL1 inhibitor atezolizumab, response rates to PD1/PDL1 pathway checkpoint inhibitors are generally quite low in HGSC, with the best objective response rates between 8% and 15% (96). Our study has identified a subset of patients with combined *RB1* and *BRCA* inactivation who demonstrate exceptional immune responses and may provide clues for the development of new immunotherapeutic strategies for HGSC that extend beyond targeting PDL1/PD1.

Our work highlights the importance of RB1 loss to treatment response and survival and focuses attention on other therapeutic opportunities in this subset of HGSC. Approximately 20% of HGSCs have a somatic loss of *RB1* assessed using genomic data (3, 27), a figure that is consistent with the IHC results obtained in the large patient cohort described here. Both approaches indicate that RB1 loss is generally clonal, enhancing its value as a therapeutic target if selective inhibitors can be identified. Although subclonal RB1 loss seems to be rare in ovarian carcinoma (0.89%), the relevance of subclonal RB1 loss should be studied in the future using full-faced tumor sections, and ideally paired primary and relapse specimens to assess clonality over time. Casein kinase 2 inhibitors have been reported to enhance the sensitivity of *RB1*-deficient TNBC and HGSC cells to carboplatin and niraparib (bioRxiv https://doi.org/10.1101/2022.11.14.516369). In addition, Aurora kinase A and B inhibition is synthetically lethal in combination with RB1 loss in breast and lung cancer cells (97–99). Irrespective of HRD status, *RB1* mutations correlate with sensitivity to WEE1 inhibition in *TP53* mutant TNBC and patient-derived HGSC xenografts (100), indicating additional treatment options that exploit RB1 inactivation in these tumors. In this study, the *BRCA1*-altered cell line AOCS7.2 with induced *RB1* knockout was more sensitive to olaparib, suggesting that RB1 loss may also predict responses to PARP inhibitors in HGSC. Most participants in the current study were diagnosed before PARP inhibitor use and *BRCA* testing was common (95% enrolled before 2013); however, our findings provide a genuine hypothesis that patients with RB1 loss may derive greater benefit from PARP inhibitors, which could be tested in newer cohorts. RB1 staining of tumor tissue by IHC is a relatively low-cost pathology-based assay that could be used in prospective studies to test whether RB1 expression is predictive of responses to PARP inhibitors, either alone or in combination with approved HRD tests.

## Supplementary Material

Supplementary Table S1 Supplementary Table S1. Details of participating Ovarian Tumor Tissue Analysis (OTTA) consortium studies and ethics approvals.

Supplementary Table S2 Supplementary Table S2. Molecular and clinical data summary of 126 patients in the Multidisciplinary Ovarian Cancer Outcomes Group (MOCOG) study.

Supplementary Table S3 Supplementary Table S3. Number of RNA-seq primary tumor samples by library type and alteration group.

Supplementary Table S4 Supplementary Table S4. Differential gene expression analysis comparing transcriptomes of tumors based on BRCA and RB1 alteration status.

Supplementary Table S5 Supplementary Table S5. Summary of cell lines used in this study.

Supplementary Table S6 Supplementary Table S6. Summary of gene alterations in cell lines.

Supplementary Table S7 Supplementary Table S7. Sequences of single guide RNAs used for CRISPR-mediated gene knockout.

Supplementary Table S8 Supplementary Table S8. Antibodies and reagents.

Supplementary Table S9 Supplementary Table S9. Primers used in this study.

Supplementary Table S10 Supplementary Table S10. Number of patients by study and histotype.

Supplementary Table S11 Supplementary Table S11. Multivariable adjusted association of molecular alterations and overall survival in HGSC and ENOC among patients with residual disease* status.

Supplementary Table S12 Supplementary Table S12. Clinical characteristics of patients with HGSC.

Supplementary Table S13 Supplementary Table S13. Clinical and molecular characteristics of ENOC.

Supplementary Table S14 Supplementary Table S14. Multivariable adjusted association of molecular alterations and overall survival in ENOC among patients with grade information.

Supplementary Table S15 Supplementary Table S15. Clinical characteristics of patients with HGSC according to RB1 and BRCA status.

Supplementary Table S16 Supplementary Table S16. Median survival according to germline BRCA1 and BRCA2 status by RB1 protein expression.

Supplementary Table S17 Supplementary Table S17. Half maximum inhibitory concentrations (IC50) for cisplatin (72 hours), paclitaxel (72 hours), or olaparib (120 hours) in HGSC cell lines with innate RB1 and/or BRCA1 alterations.

Supplementary Table S18 Supplementary Table S18. Half maximum inhibitory concentrations (IC50) for cisplatin, paclitaxel and olaparib in cell lines after CRISPR/Cas-9 mediated RB1 knockout.

Supplementary Table S19 Supplementary Table S19. Relative expression of BRCA1 and RB1 by qPCR in AOCS30 CRISPR knockout model.

Supplementary Table S20 Supplementary Table S20. Immune cell type (LM22) absolute abundance in 126 primary HGSC tumors with CIBERSORTx.

Supplementary Figure S1 Supplementary Figure S1. Patients and tumor samples analyzed in this study.

Supplementary Figure S2 Supplementary Figure S2. Representative immunohistochemical RB1 staining patterns.

Supplementary Figure S3 Supplementary Figure S3. Overall survival by combined RB1 and p53 expression in ENOC, and RB1 protein expression and BRCA status in HGSC.

Supplementary Figure S4 Supplementary Figure S4. HGSC cell lines with innate RB1 and/or BRCA1 alterations.

Supplementary Figure S5 Supplementary Figure S5. Cell proliferation and cell cycle distribution of HGSC cell lines with RB1 knockout.

Supplementary Figure S6 Supplementary Figure S6. Mutational signatures in homologous recombination deficiency and RB1 subgroups.

Supplementary Figure S7 Supplementary Figure S7. Genomic and clinical characteristics by combined homologous recombination deficiency and RB1 status.

Supplementary Figure S8 Supplementary Figure S8. Gene alterations across BRCA and RB1 altered subgroups.

Supplementary Figure S9 Supplementary Figure S9. Bars indicate the number of differentially expressed genes (Benjamini-Hochberg adjusted P value < 0.05) between HGSC tumors grouped by HRD and/or RB1 status as shown.

Supplementary Figure S10 Supplementary Figure S10. Immune cell subsets inferred from gene expression data by combined homologous recombination deficiency and RB1 status.
