# Supplementary material for: Concurrent RB1 Loss and BRCA Deficiency Predicts Enhanced Immunologic Response and Long-term Survival in Tubo-ovarian High-grade Serous Carcinoma
Source: Clin Cancer Res. 2024 Jun 5;30(16):3481–98. doi: 10.1158/1078-0432.CCR-23-3552 (PMC11325151; doi:10.1158/1078-0432.CCR-23-3552)
Supplement: Supplementary Figure S1 — Patients and tumor samples analyzed in this study. [file ccr-23-3552_supplementary_figure_s1_suppsf1.pptx]

## Slide 1
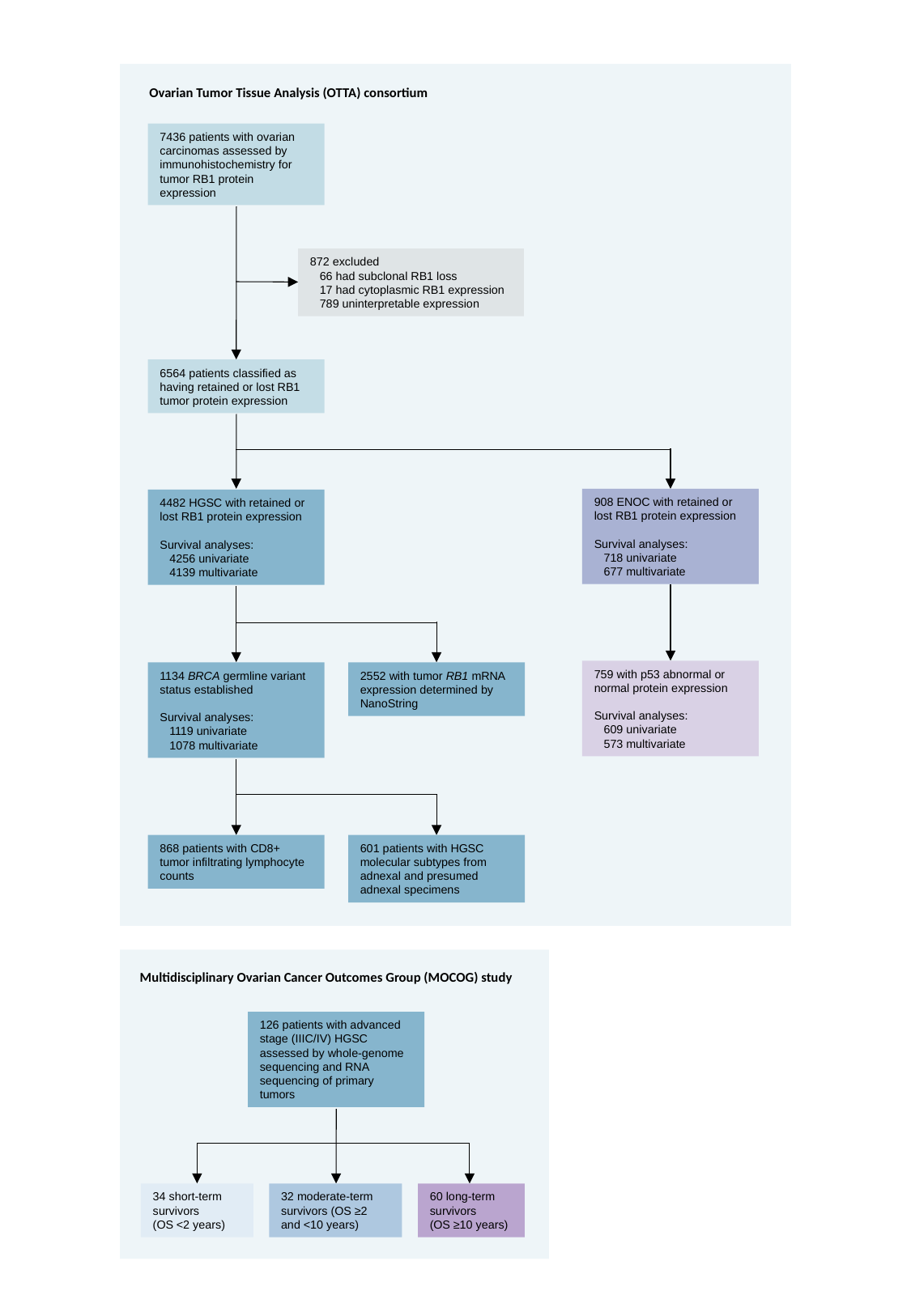

Ovarian Tumor Tissue Analysis (OTTA) consortium
7436 patients with ovarian carcinomas assessed by immunohistochemistry for tumor RB1 protein expression
872 excluded
 66 had subclonal RB1 loss
 17 had cytoplasmic RB1 expression
 789 uninterpretable expression
6564 patients classified as having retained or lost RB1 tumor protein expression
908 ENOC with retained or lost RB1 protein expression
Survival analyses:
 718 univariate
 677 multivariate
4482 HGSC with retained or lost RB1 protein expression
Survival analyses:
 4256 univariate
 4139 multivariate
759 with p53 abnormal or normal protein expression
Survival analyses:
 609 univariate
 573 multivariate
2552 with tumor RB1 mRNA expression determined by NanoString
1134 BRCA germline variant status established
Survival analyses:
 1119 univariate
 1078 multivariate
868 patients with CD8+ tumor infiltrating lymphocyte counts
601 patients with HGSC molecular subtypes from adnexal and presumed adnexal specimens
Multidisciplinary Ovarian Cancer Outcomes Group (MOCOG) study
126 patients with advanced stage (IIIC/IV) HGSC assessed by whole-genome sequencing and RNA sequencing of primary tumors
34 short-term survivors
(OS <2 years)
32 moderate-term
survivors (OS ≥2 and <10 years)
60 long-term
survivors
(OS ≥10 years)

## Slide 2
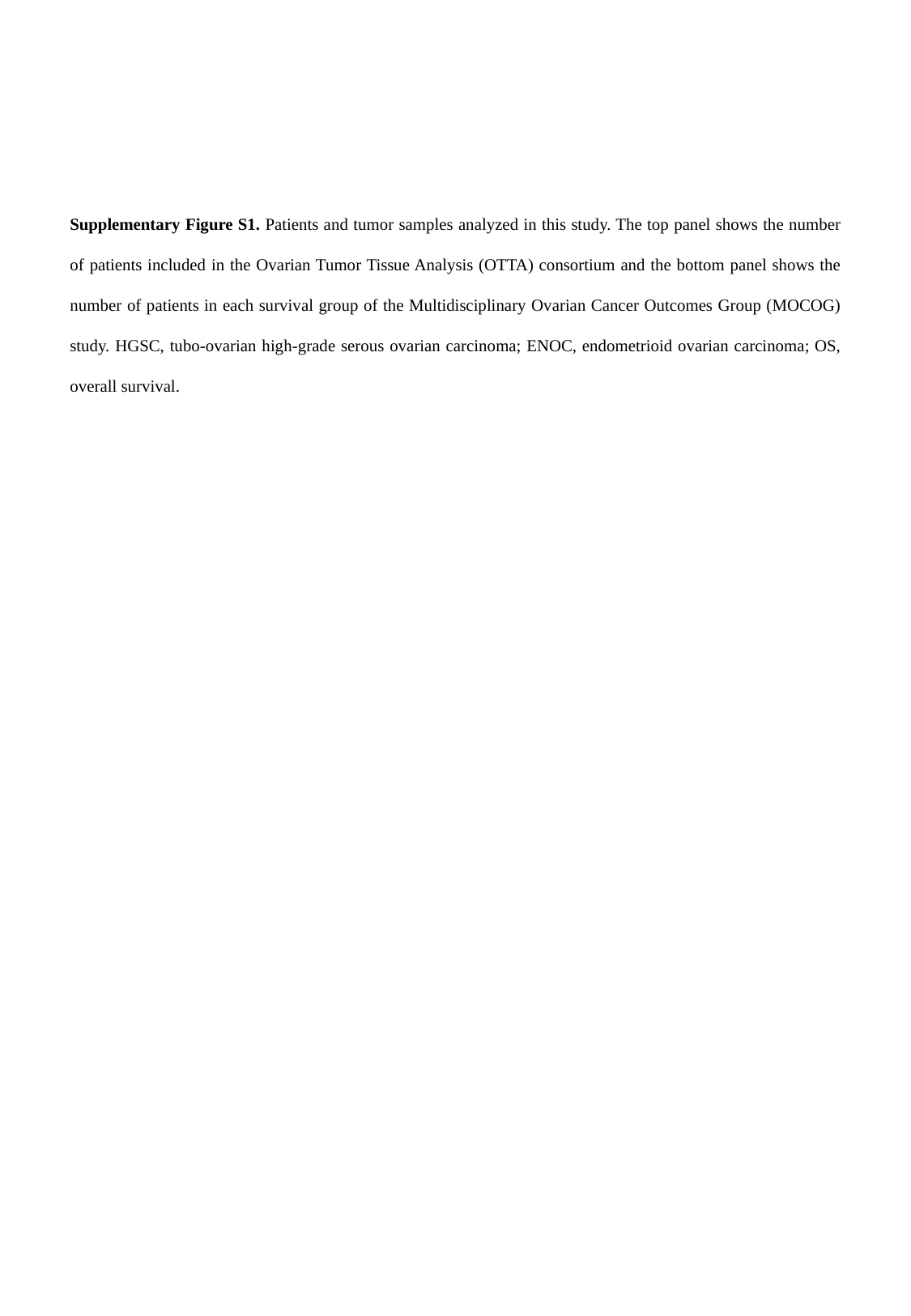

Supplementary Figure S1. Patients and tumor samples analyzed in this study. The top panel shows the number of patients included in the Ovarian Tumor Tissue Analysis (OTTA) consortium and the bottom panel shows the number of patients in each survival group of the Multidisciplinary Ovarian Cancer Outcomes Group (MOCOG) study. HGSC, tubo-ovarian high-grade serous ovarian carcinoma; ENOC, endometrioid ovarian carcinoma; OS, overall survival.
