# Supplementary material for: Concurrent RB1 Loss and BRCA Deficiency Predicts Enhanced Immunologic Response and Long-term Survival in Tubo-ovarian High-grade Serous Carcinoma
Source: Clin Cancer Res. 2024 Jun 5;30(16):3481–98. doi: 10.1158/1078-0432.CCR-23-3552 (PMC11325151; doi:10.1158/1078-0432.CCR-23-3552)
Supplement: Supplementary Figure S2 — Representative immunohistochemical RB1 staining patterns. [file ccr-23-3552_supplementary_figure_s2_suppsf2.docx]

| 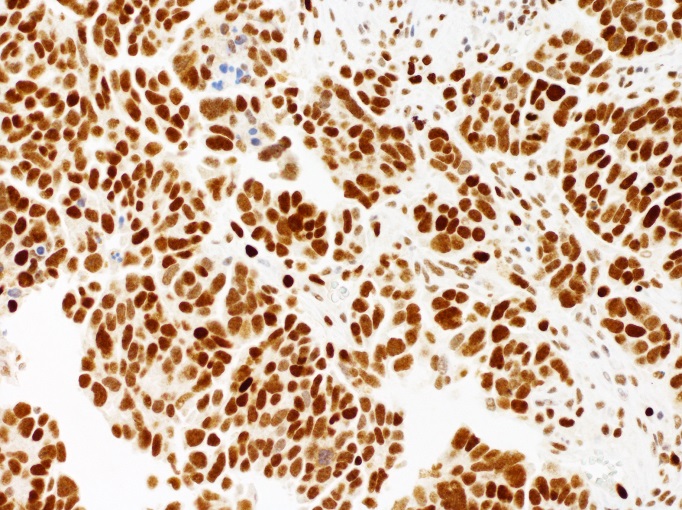 | 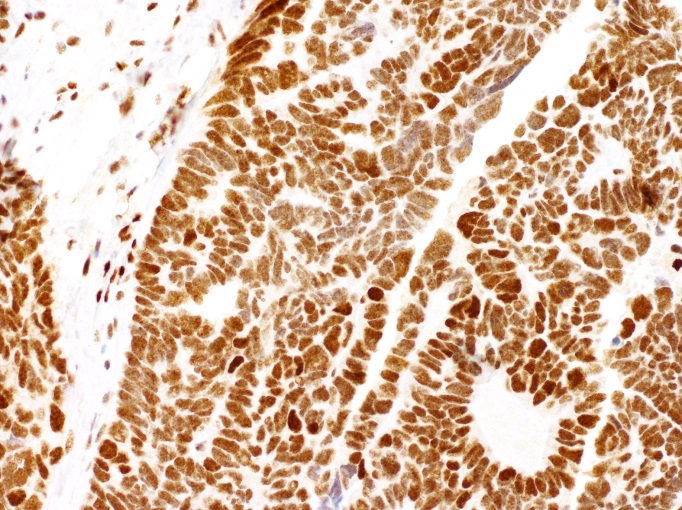 |
| --- | --- |
| Score 1 = present | Score 1 = present |
| 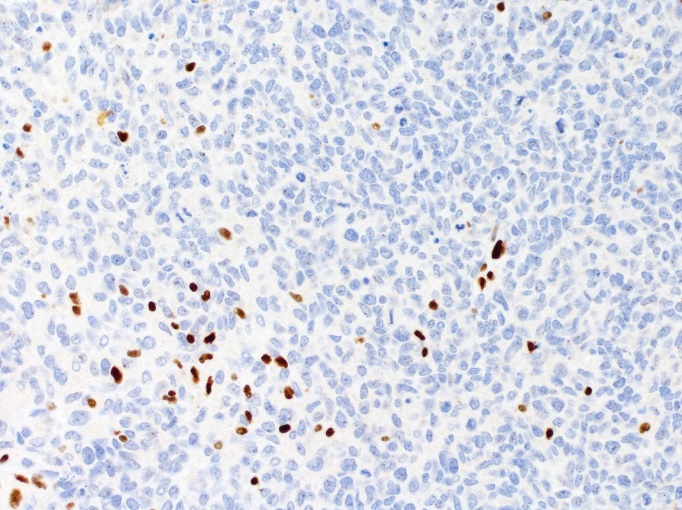 | 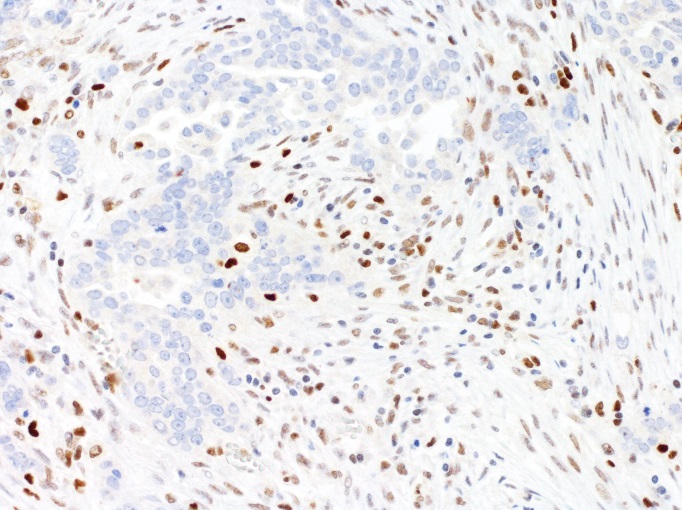 |
| Score 0 = absent | Score 0 = absent |
| 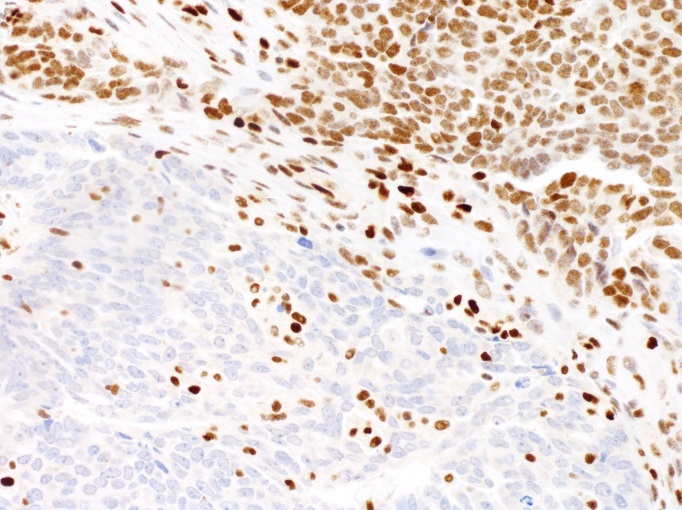 | 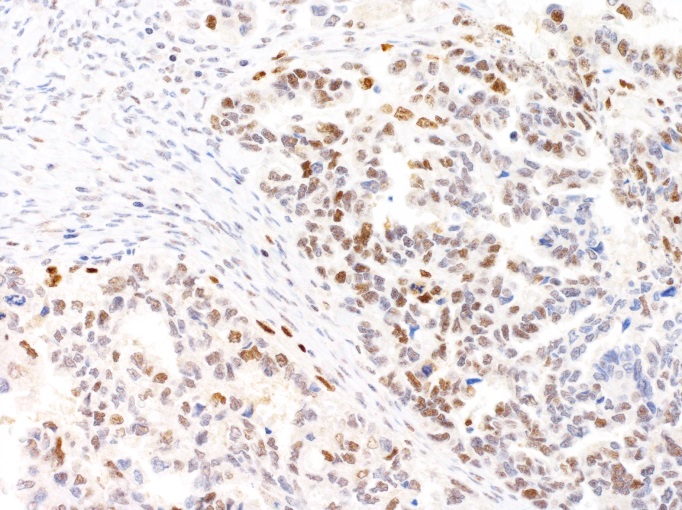 |
| Score 2 = subclonal loss | Score 1 = present (although heterogeneously reduced) |
| 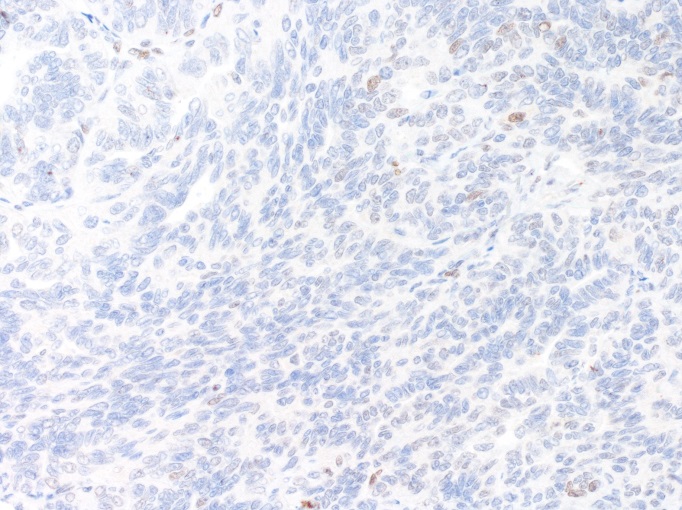 |  |
| Score 8 = no staining and lack of internal control |  |

**Supplementary Figure S2.** Representative immunohistochemical RB1 staining patterns. Images show examples of tumors with RB1 protein present, absent, subclonal loss, present heterogeneously, and absent but lacking adjacent internal control.
