# Supplementary material for: Concurrent RB1 Loss and BRCA Deficiency Predicts Enhanced Immunologic Response and Long-term Survival in Tubo-ovarian High-grade Serous Carcinoma
Source: Clin Cancer Res. 2024 Jun 5;30(16):3481–98. doi: 10.1158/1078-0432.CCR-23-3552 (PMC11325151; doi:10.1158/1078-0432.CCR-23-3552)
Supplement: Supplementary Figure S3 — Overall survival by combined RB1 and p53 expression in ENOC, and RB1 protein expression and BRCA status in HGSC. [file ccr-23-3552_supplementary_figure_s3_suppsf3.pptx]

## Slide 1
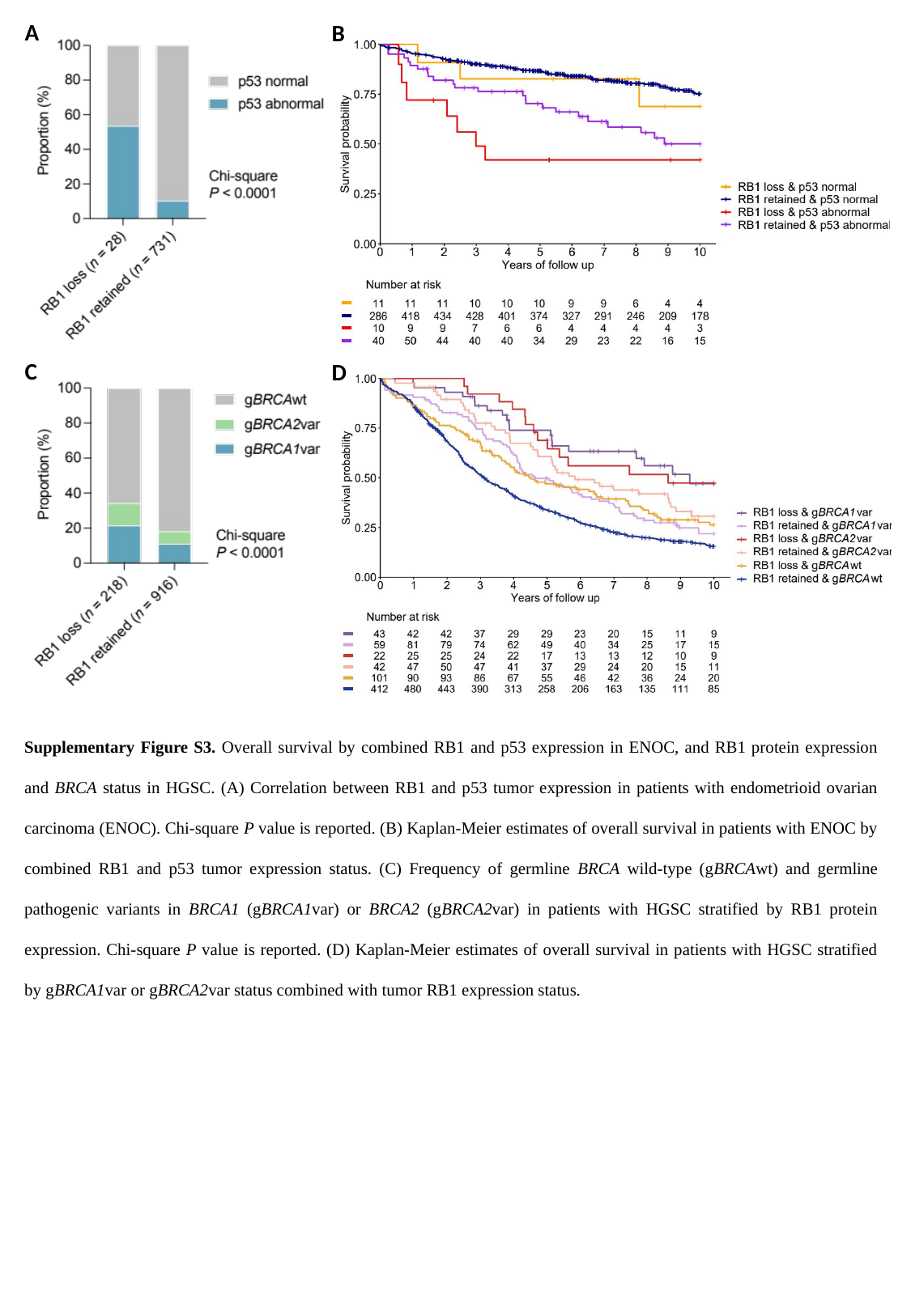

A
B
C
D
Supplementary Figure S3. Overall survival by combined RB1 and p53 expression in ENOC, and RB1 protein expression and BRCA status in HGSC. (A) Correlation between RB1 and p53 tumor expression in patients with endometrioid ovarian carcinoma (ENOC). Chi-square P value is reported. (B) Kaplan-Meier estimates of overall survival in patients with ENOC by combined RB1 and p53 tumor expression status. (C) Frequency of germline BRCA wild-type (gBRCAwt) and germline pathogenic variants in BRCA1 (gBRCA1var) or BRCA2 (gBRCA2var) in patients with HGSC stratified by RB1 protein expression. Chi-square P value is reported. (D) Kaplan-Meier estimates of overall survival in patients with HGSC stratified by gBRCA1var or gBRCA2var status combined with tumor RB1 expression status.
