# Supplementary material for: Concurrent RB1 Loss and BRCA Deficiency Predicts Enhanced Immunologic Response and Long-term Survival in Tubo-ovarian High-grade Serous Carcinoma
Source: Clin Cancer Res. 2024 Jun 5;30(16):3481–98. doi: 10.1158/1078-0432.CCR-23-3552 (PMC11325151; doi:10.1158/1078-0432.CCR-23-3552)
Supplement: Supplementary Figure S4 — HGSC cell lines with innate RB1 and/or BRCA1 alterations. [file ccr-23-3552_supplementary_figure_s4_suppsf4.pptx]

## Slide 1
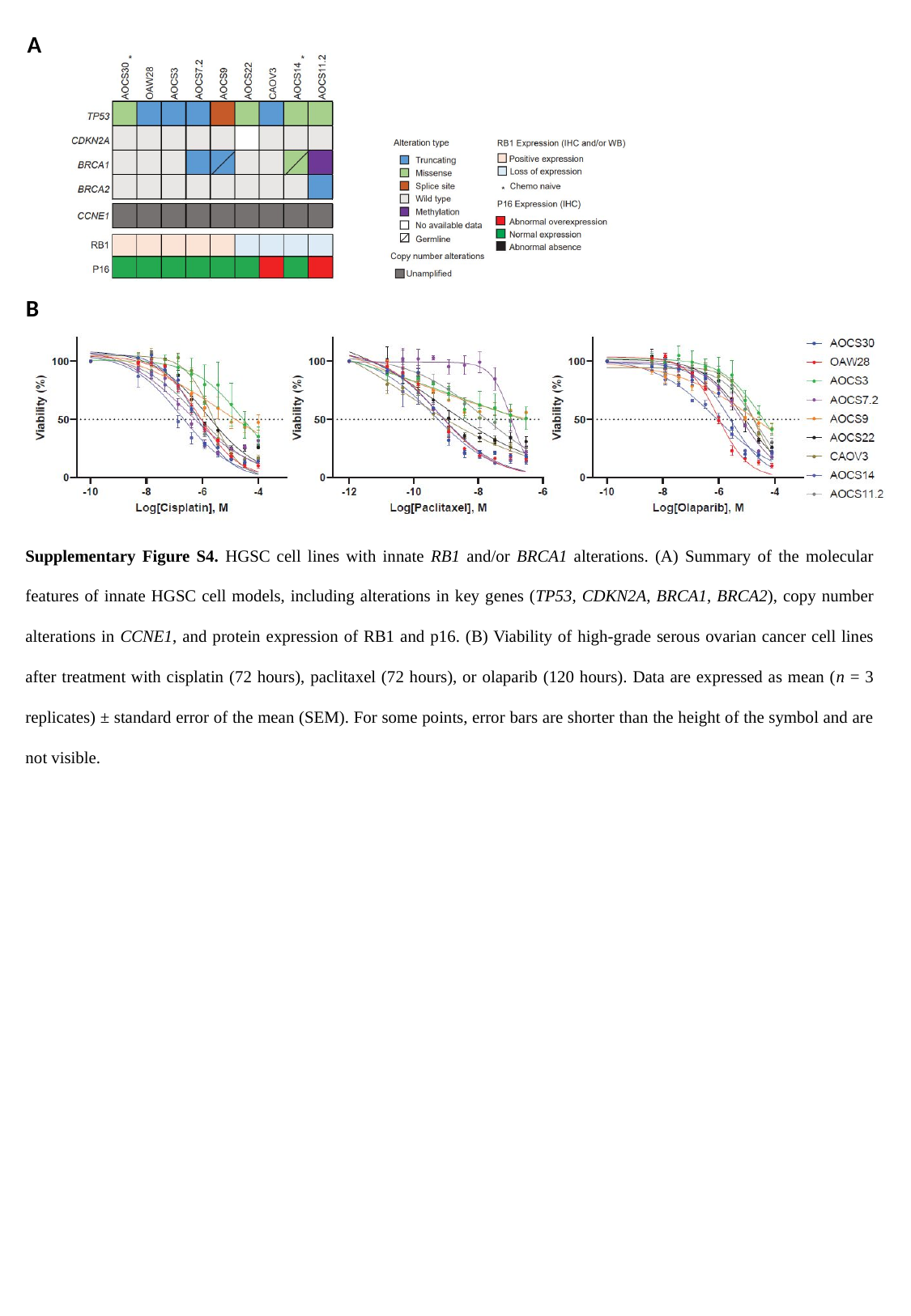

A
B
Supplementary Figure S4. HGSC cell lines with innate RB1 and/or BRCA1 alterations. (A) Summary of the molecular features of innate HGSC cell models, including alterations in key genes (TP53, CDKN2A, BRCA1, BRCA2), copy number alterations in CCNE1, and protein expression of RB1 and p16. (B) Viability of high-grade serous ovarian cancer cell lines after treatment with cisplatin (72 hours), paclitaxel (72 hours), or olaparib (120 hours). Data are expressed as mean (n = 3 replicates) ± standard error of the mean (SEM). For some points, error bars are shorter than the height of the symbol and are not visible.
