# Supplementary material for: Concurrent RB1 Loss and BRCA Deficiency Predicts Enhanced Immunologic Response and Long-term Survival in Tubo-ovarian High-grade Serous Carcinoma
Source: Clin Cancer Res. 2024 Jun 5;30(16):3481–98. doi: 10.1158/1078-0432.CCR-23-3552 (PMC11325151; doi:10.1158/1078-0432.CCR-23-3552)
Supplement: Supplementary Figure S5 — Cell proliferation and cell cycle distribution of HGSC cell lines with RB1 knockout. [file ccr-23-3552_supplementary_figure_s5_suppsf5.pptx]

## Slide 1
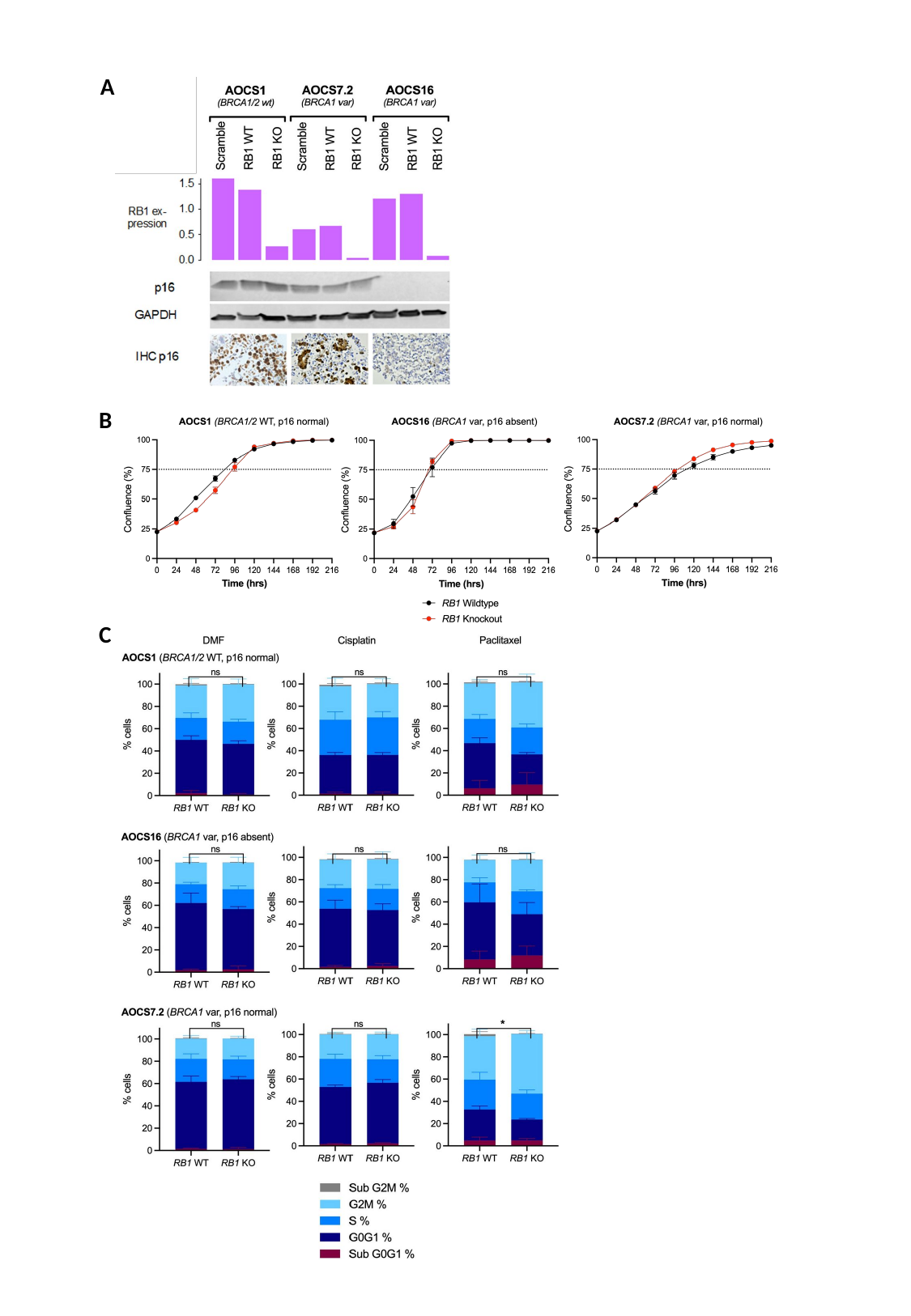

A
B
C

## Slide 2
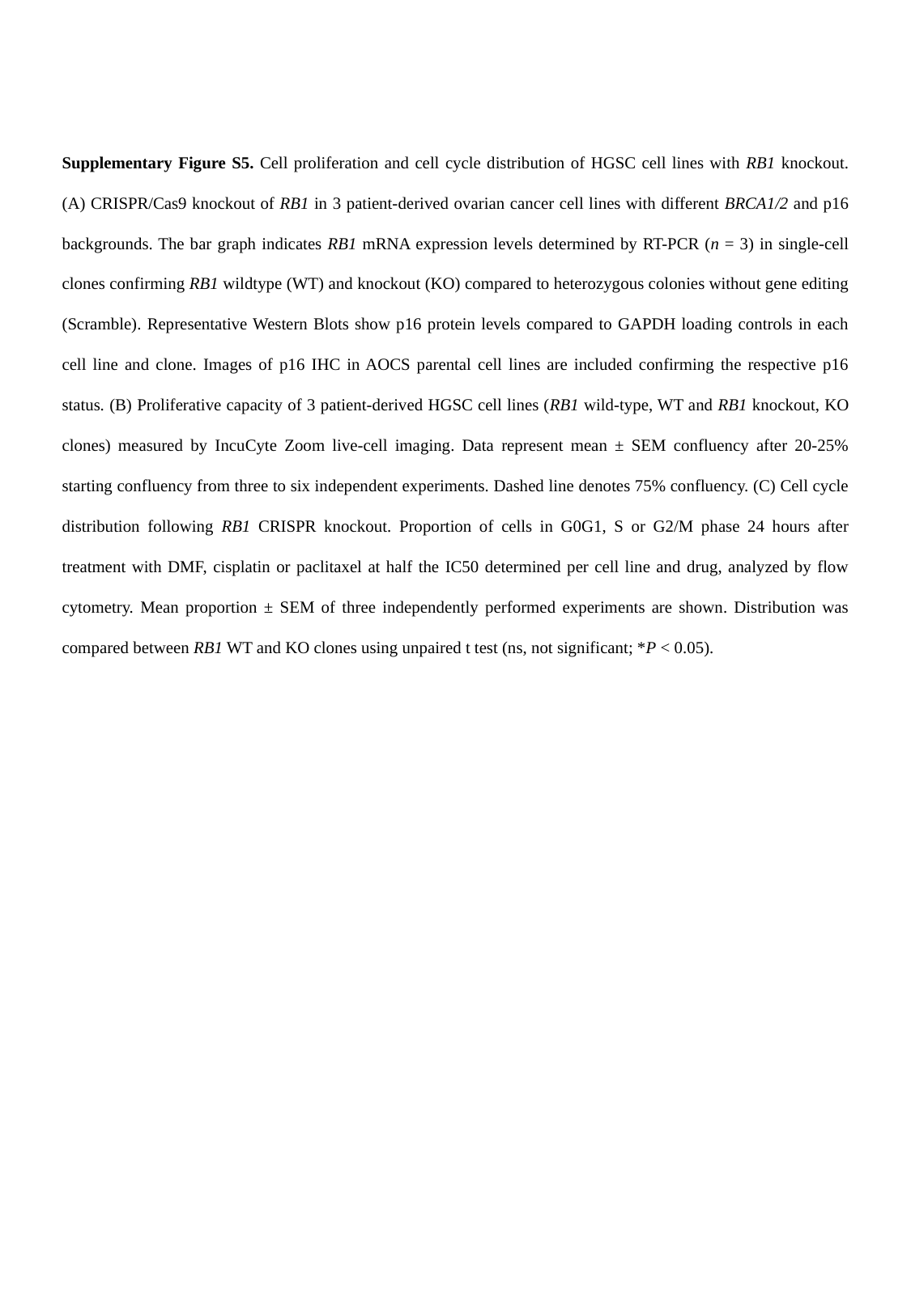

Supplementary Figure S5. Cell proliferation and cell cycle distribution of HGSC cell lines with RB1 knockout. (A) CRISPR/Cas9 knockout of RB1 in 3 patient-derived ovarian cancer cell lines with different BRCA1/2 and p16 backgrounds. The bar graph indicates RB1 mRNA expression levels determined by RT-PCR (n = 3) in single-cell clones confirming RB1 wildtype (WT) and knockout (KO) compared to heterozygous colonies without gene editing (Scramble). Representative Western Blots show p16 protein levels compared to GAPDH loading controls in each cell line and clone. Images of p16 IHC in AOCS parental cell lines are included confirming the respective p16 status. (B) Proliferative capacity of 3 patient-derived HGSC cell lines (RB1 wild-type, WT and RB1 knockout, KO clones) measured by IncuCyte Zoom live-cell imaging. Data represent mean ± SEM confluency after 20-25% starting confluency from three to six independent experiments. Dashed line denotes 75% confluency. (C) Cell cycle distribution following RB1 CRISPR knockout. Proportion of cells in G0G1, S or G2/M phase 24 hours after treatment with DMF, cisplatin or paclitaxel at half the IC50 determined per cell line and drug, analyzed by flow cytometry. Mean proportion ± SEM of three independently performed experiments are shown. Distribution was compared between RB1 WT and KO clones using unpaired t test (ns, not significant; *P < 0.05).
