# Supplementary material for: Concurrent RB1 Loss and BRCA Deficiency Predicts Enhanced Immunologic Response and Long-term Survival in Tubo-ovarian High-grade Serous Carcinoma
Source: Clin Cancer Res. 2024 Jun 5;30(16):3481–98. doi: 10.1158/1078-0432.CCR-23-3552 (PMC11325151; doi:10.1158/1078-0432.CCR-23-3552)
Supplement: Supplementary Figure S6 — Mutational signatures in homologous recombination deficiency and RB1 subgroups. [file ccr-23-3552_supplementary_figure_s6_suppsf6.pptx]

## Slide 1
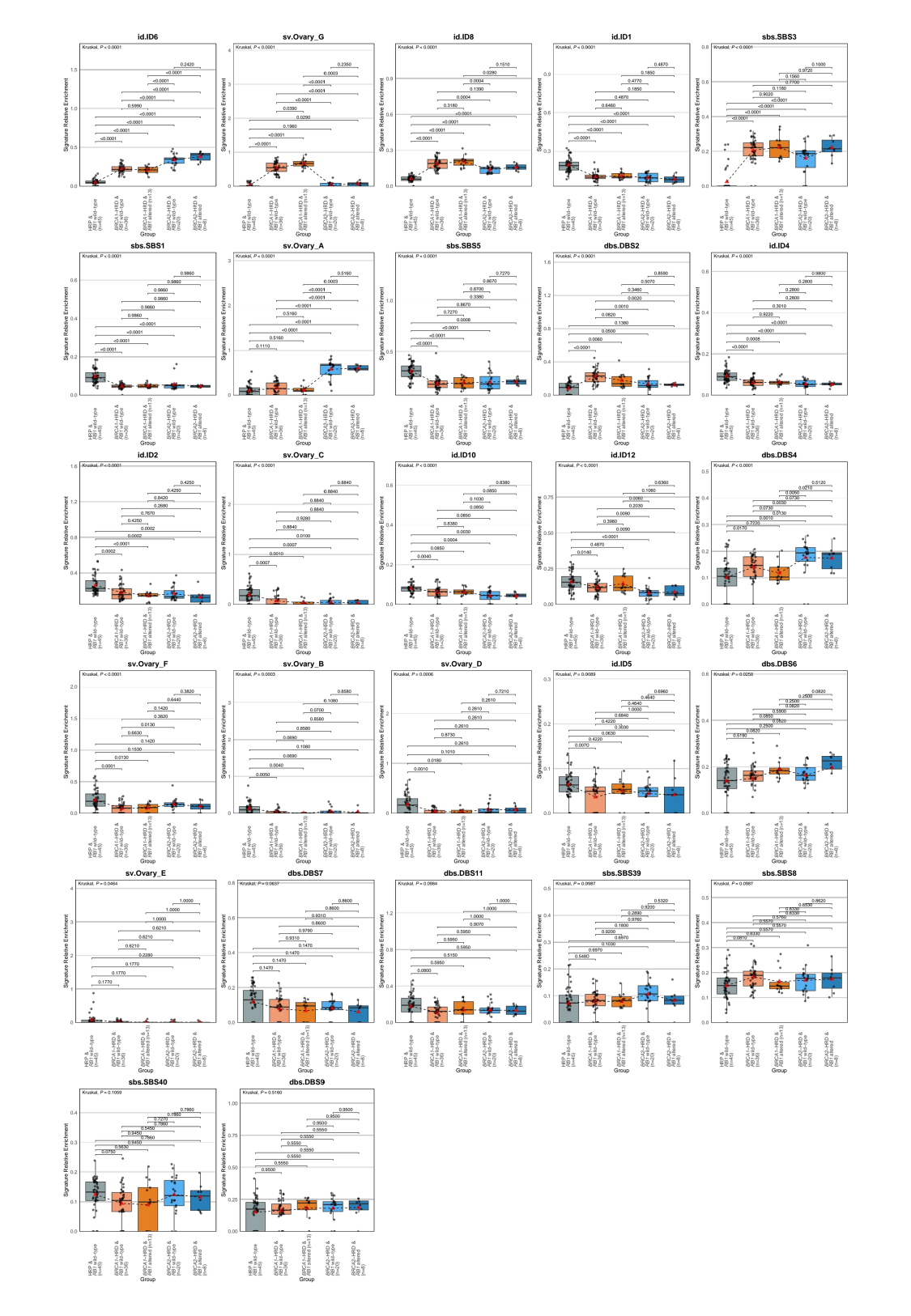

## Slide 2
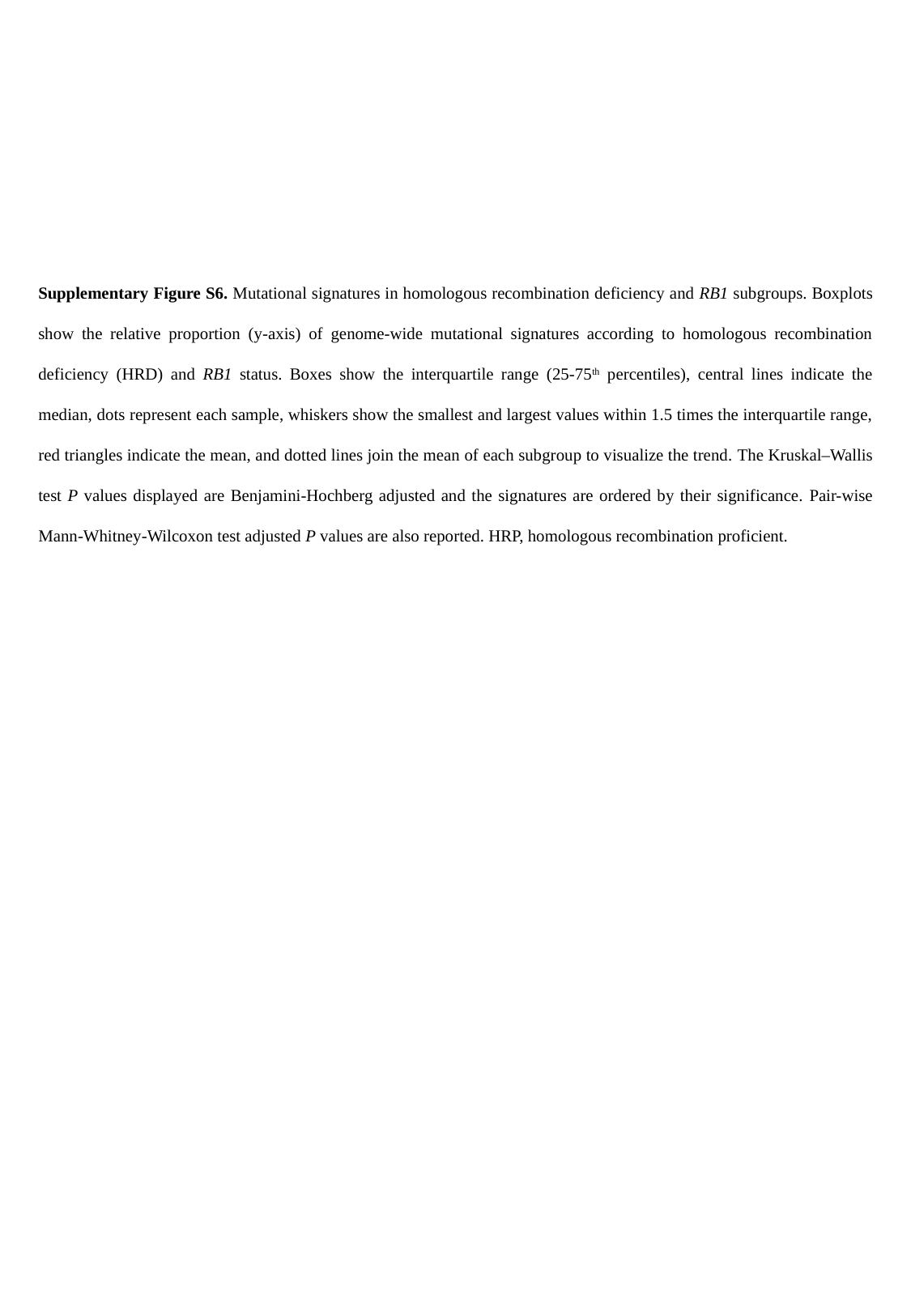

Supplementary Figure S6. Mutational signatures in homologous recombination deficiency and RB1 subgroups. Boxplots show the relative proportion (y-axis) of genome-wide mutational signatures according to homologous recombination deficiency (HRD) and RB1 status. Boxes show the interquartile range (25-75th percentiles), central lines indicate the median, dots represent each sample, whiskers show the smallest and largest values within 1.5 times the interquartile range, red triangles indicate the mean, and dotted lines join the mean of each subgroup to visualize the trend. ﻿The Kruskal–Wallis test P values displayed are Benjamini-Hochberg adjusted and the signatures are ordered by their significance. ﻿Pair-wise Mann-Whitney-Wilcoxon test adjusted P values are also reported. HRP, homologous recombination proficient.
