# Supplementary material for: Concurrent RB1 Loss and BRCA Deficiency Predicts Enhanced Immunologic Response and Long-term Survival in Tubo-ovarian High-grade Serous Carcinoma
Source: Clin Cancer Res. 2024 Jun 5;30(16):3481–98. doi: 10.1158/1078-0432.CCR-23-3552 (PMC11325151; doi:10.1158/1078-0432.CCR-23-3552)
Supplement: Supplementary Figure S7 — Genomic and clinical characteristics by combined homologous recombination deficiency and RB1 status. [file ccr-23-3552_supplementary_figure_s7_suppsf7.pptx]

## Slide 1
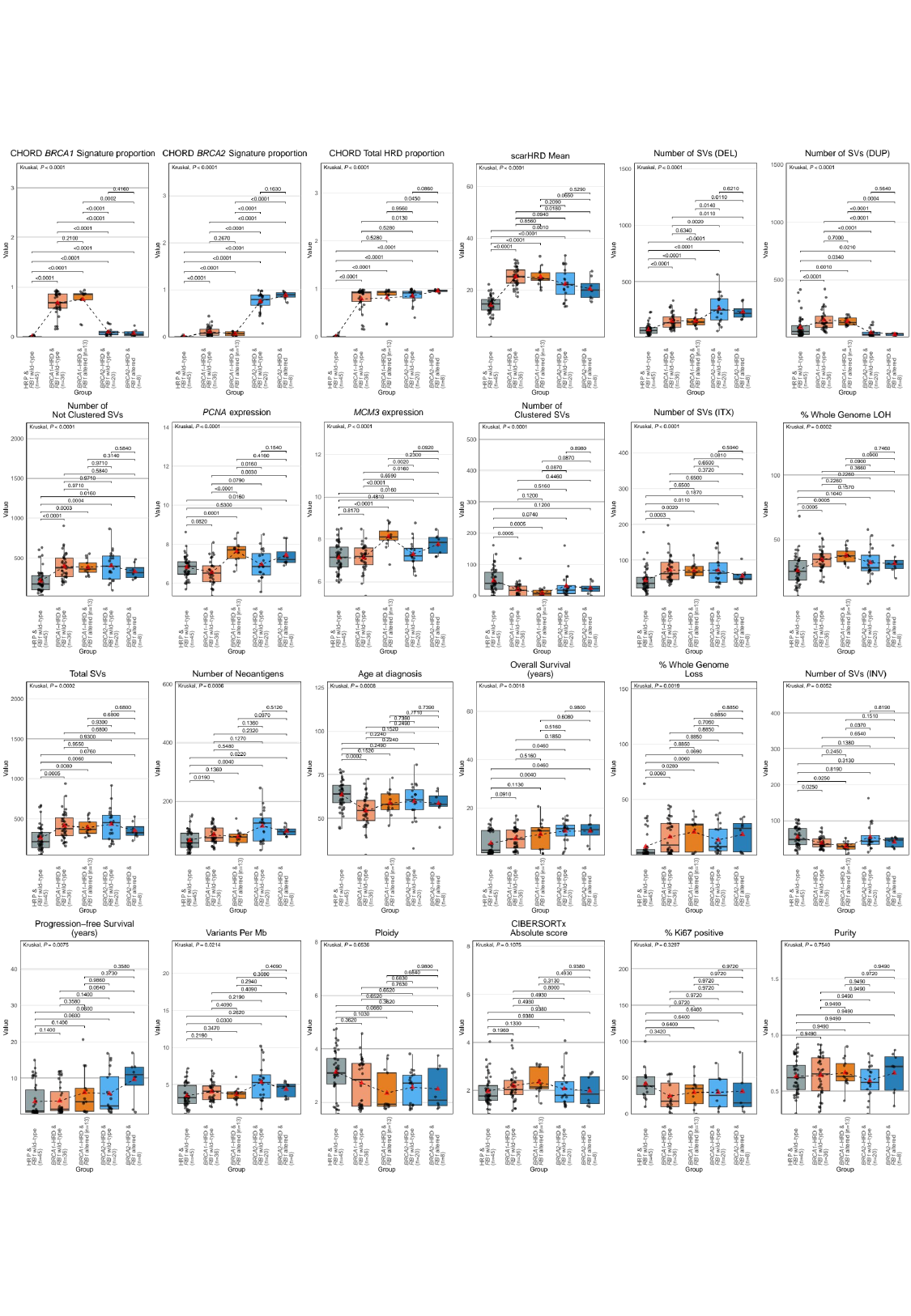

## Slide 2
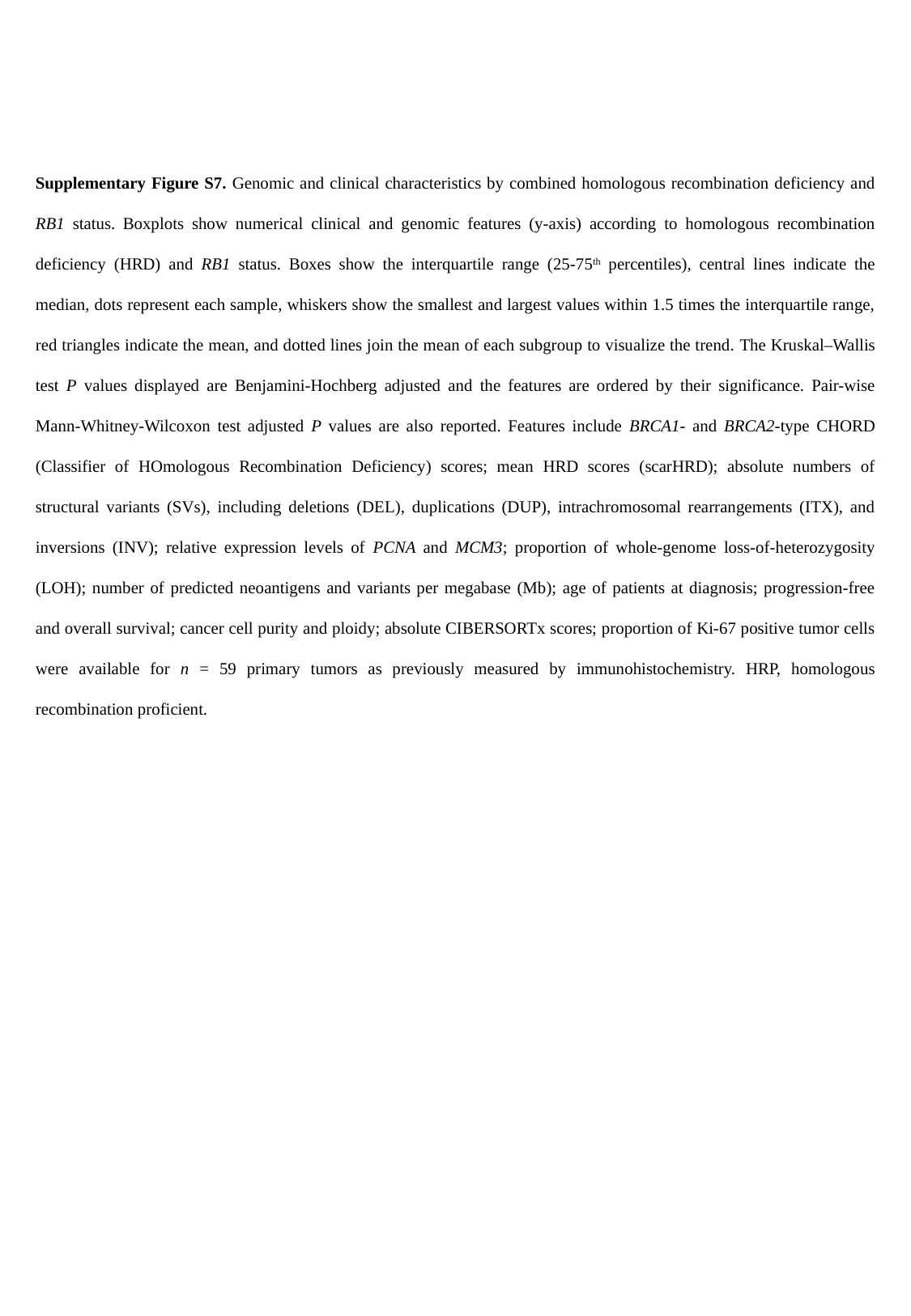

Supplementary Figure S7. Genomic and clinical characteristics by combined homologous recombination deficiency and RB1 status. Boxplots ﻿show numerical clinical and genomic features (y-axis) according to homologous recombination deficiency (HRD) and RB1 status. Boxes show the interquartile range (25-75th percentiles), central lines indicate the median, dots represent each sample, whiskers show the smallest and largest values within 1.5 times the interquartile range, red triangles indicate the mean, and dotted lines join the mean of each subgroup to visualize the trend. ﻿The Kruskal–Wallis test P values displayed are Benjamini-Hochberg adjusted and the features are ordered by their significance. Pair-wise Mann-Whitney-Wilcoxon test adjusted P values are also reported. Features include BRCA1- and BRCA2-type CHORD (Classifier of HOmologous Recombination Deficiency) scores; mean HRD scores (scarHRD); absolute numbers of structural variants (SVs), including deletions (DEL), duplications (DUP), intrachromosomal rearrangements (ITX), and inversions (INV); relative expression levels of PCNA and MCM3; proportion of whole-genome loss-of-heterozygosity (LOH); number of predicted neoantigens and variants per megabase (Mb); age of patients at diagnosis; progression-free and overall survival; cancer cell purity and ploidy; absolute CIBERSORTx scores; proportion of Ki-67 positive tumor cells were available for n = 59 primary tumors as previously measured by immunohistochemistry. HRP, homologous recombination proficient.
