# Supplementary material for: Concurrent RB1 Loss and BRCA Deficiency Predicts Enhanced Immunologic Response and Long-term Survival in Tubo-ovarian High-grade Serous Carcinoma
Source: Clin Cancer Res. 2024 Jun 5;30(16):3481–98. doi: 10.1158/1078-0432.CCR-23-3552 (PMC11325151; doi:10.1158/1078-0432.CCR-23-3552)
Supplement: Supplementary Figure S8 — Gene alterations across BRCA and RB1 altered subgroups. [file ccr-23-3552_supplementary_figure_s8_suppsf8.pptx]

## Slide 1
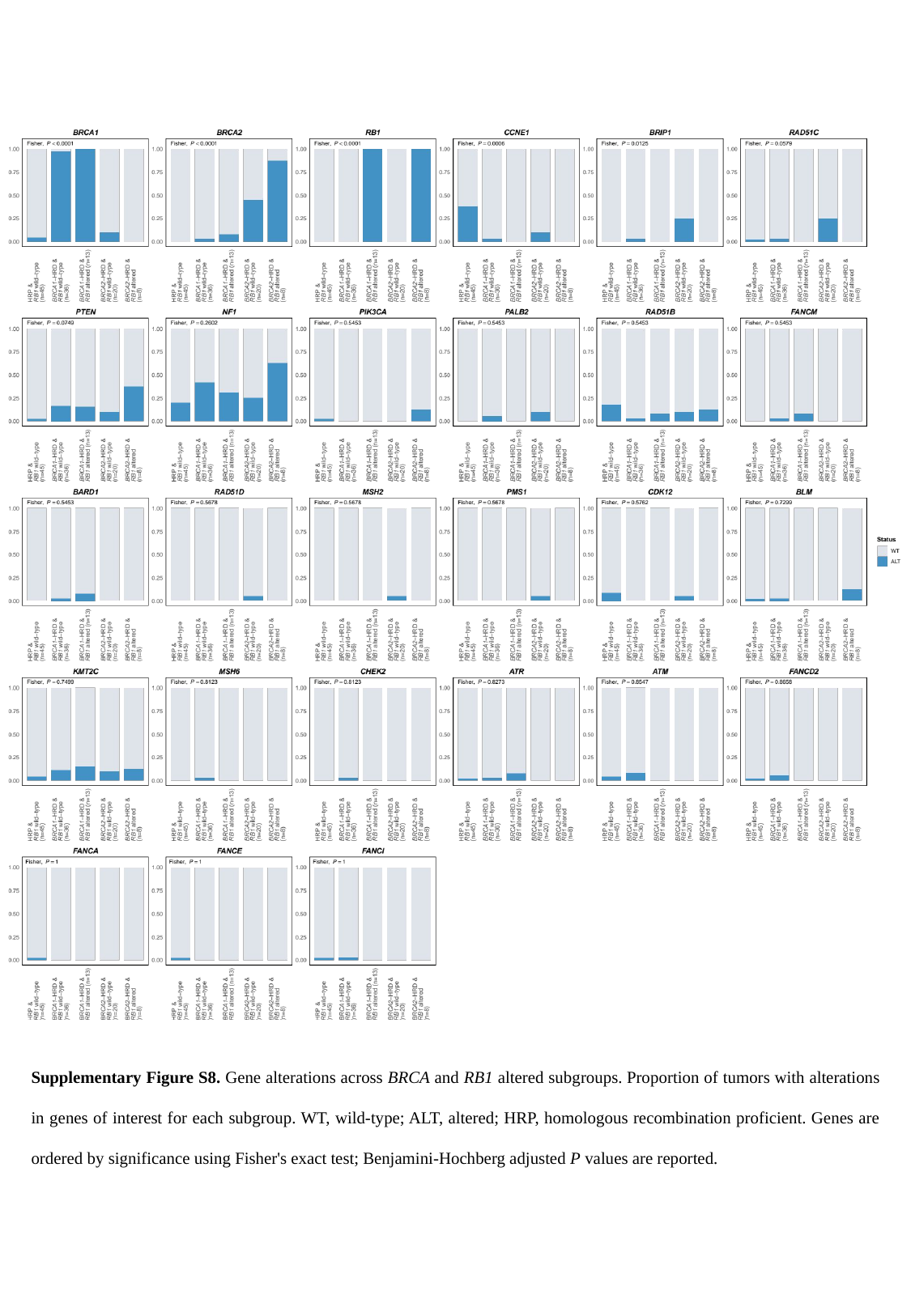

Supplementary Figure S8. Gene alterations across BRCA and RB1 altered subgroups. Proportion of tumors with alterations in genes of interest for each subgroup. WT, wild-type; ALT, altered; HRP, homologous recombination proficient. Genes are ordered by significance using Fisher's exact test; Benjamini-Hochberg adjusted P values are reported.
