# Supplementary material for: Concurrent RB1 Loss and BRCA Deficiency Predicts Enhanced Immunologic Response and Long-term Survival in Tubo-ovarian High-grade Serous Carcinoma
Source: Clin Cancer Res. 2024 Jun 5;30(16):3481–98. doi: 10.1158/1078-0432.CCR-23-3552 (PMC11325151; doi:10.1158/1078-0432.CCR-23-3552)
Supplement: Supplementary Figure S9 — Bars indicate the number of differentially expressed genes (Benjamini-Hochberg adjusted P value < 0.05) between HGSC tumors grouped by HRD and/or RB1 status as shown. [file ccr-23-3552_supplementary_figure_s9_suppsf9.pptx]

## Slide 1
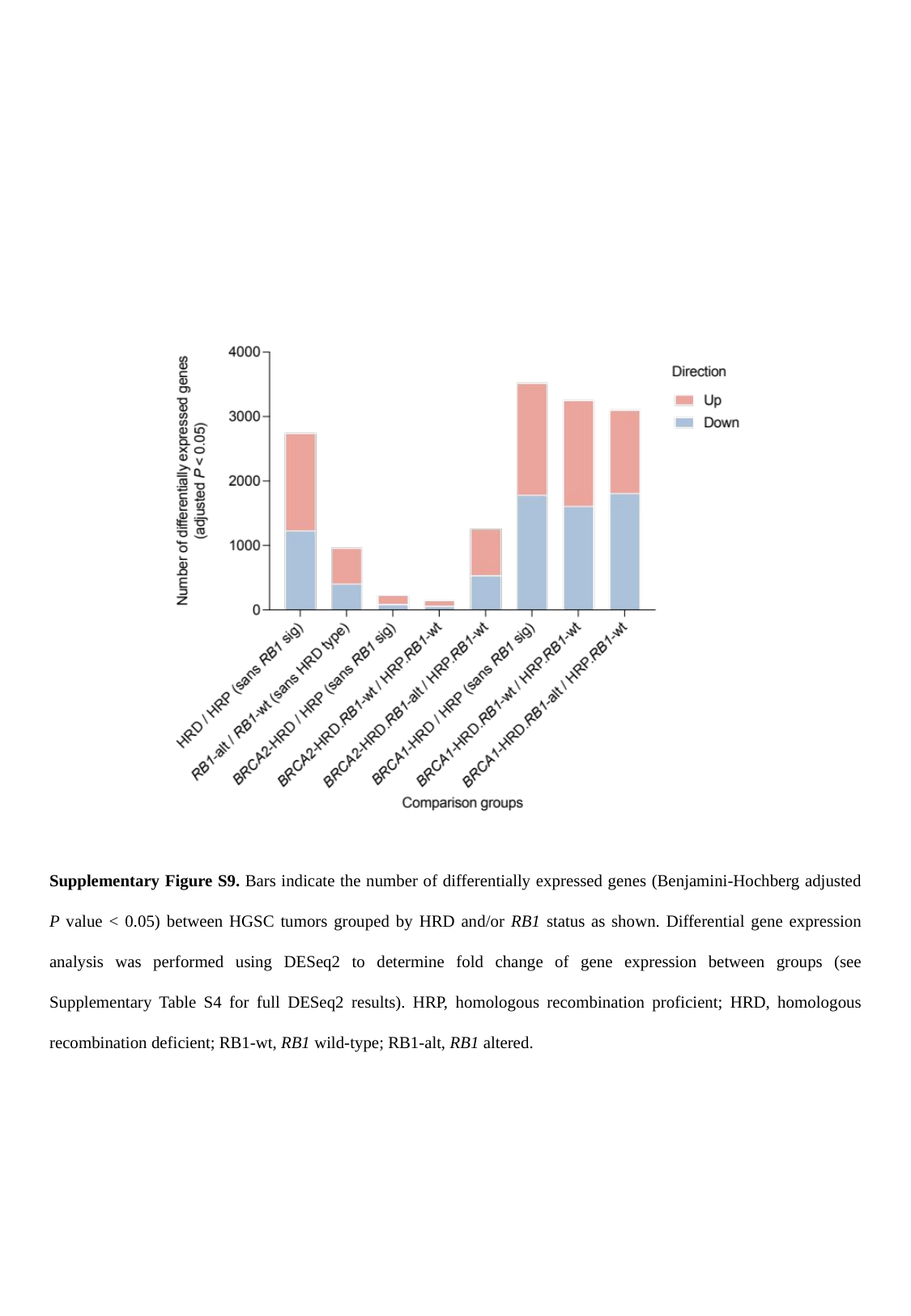

Supplementary Figure S9. Bars indicate the number of differentially expressed genes (Benjamini-Hochberg adjusted P value < 0.05) between HGSC tumors grouped by HRD and/or RB1 status as shown. Differential gene expression analysis was performed using DESeq2 to determine fold change of gene expression between groups (see Supplementary Table S4 for full DESeq2 results). HRP, homologous recombination proficient; HRD, homologous recombination deficient; RB1-wt, RB1 wild-type; RB1-alt, RB1 altered.
