# Supplementary material for: Concurrent RB1 Loss and BRCA Deficiency Predicts Enhanced Immunologic Response and Long-term Survival in Tubo-ovarian High-grade Serous Carcinoma
Source: Clin Cancer Res. 2024 Jun 5;30(16):3481–98. doi: 10.1158/1078-0432.CCR-23-3552 (PMC11325151; doi:10.1158/1078-0432.CCR-23-3552)
Supplement: Supplementary Figure S10 — Immune cell subsets inferred from gene expression data by combined homologous recombination deficiency and RB1 status. [file ccr-23-3552_supplementary_figure_s10_suppsf10.pptx]

## Slide 1
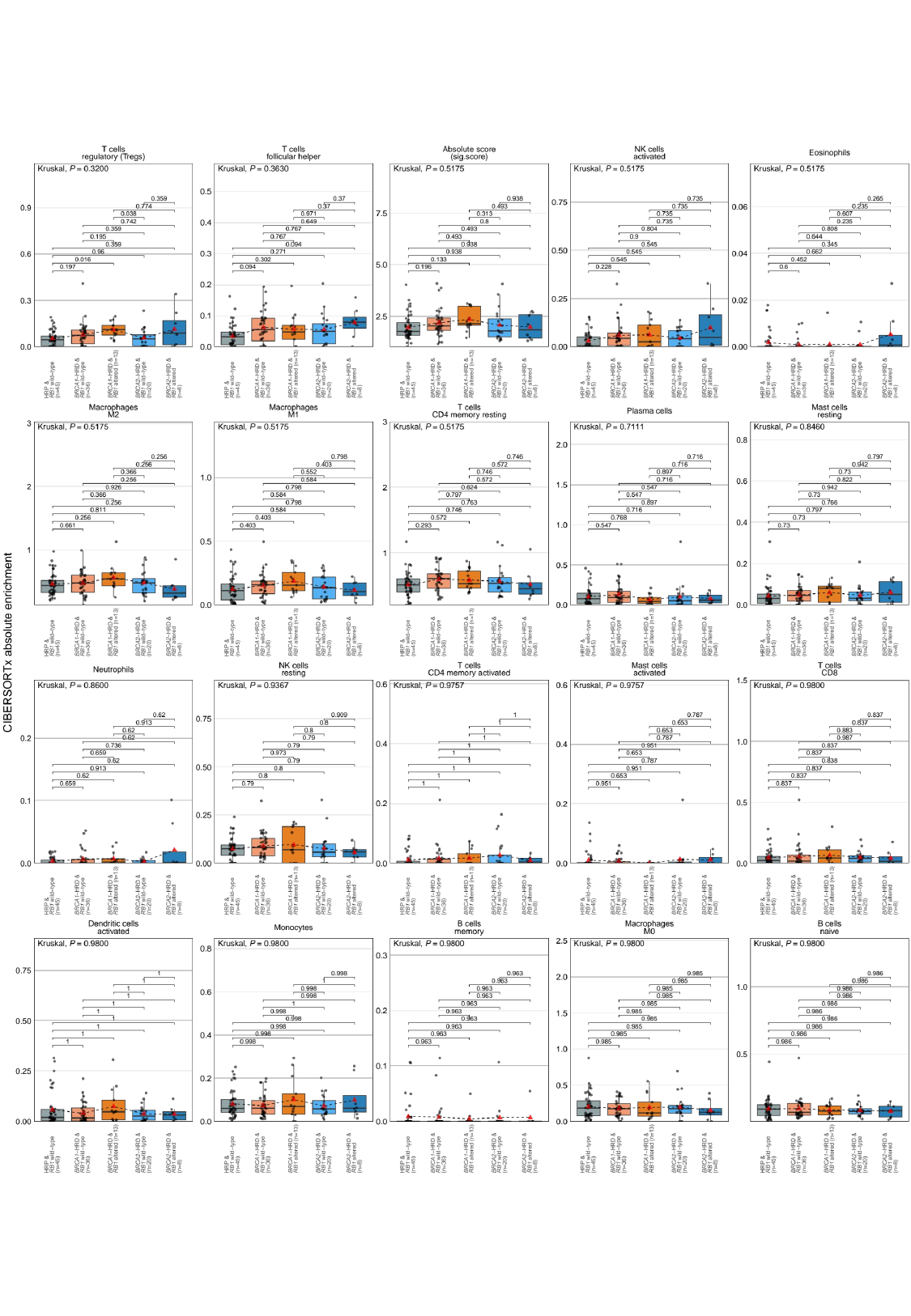

## Slide 2
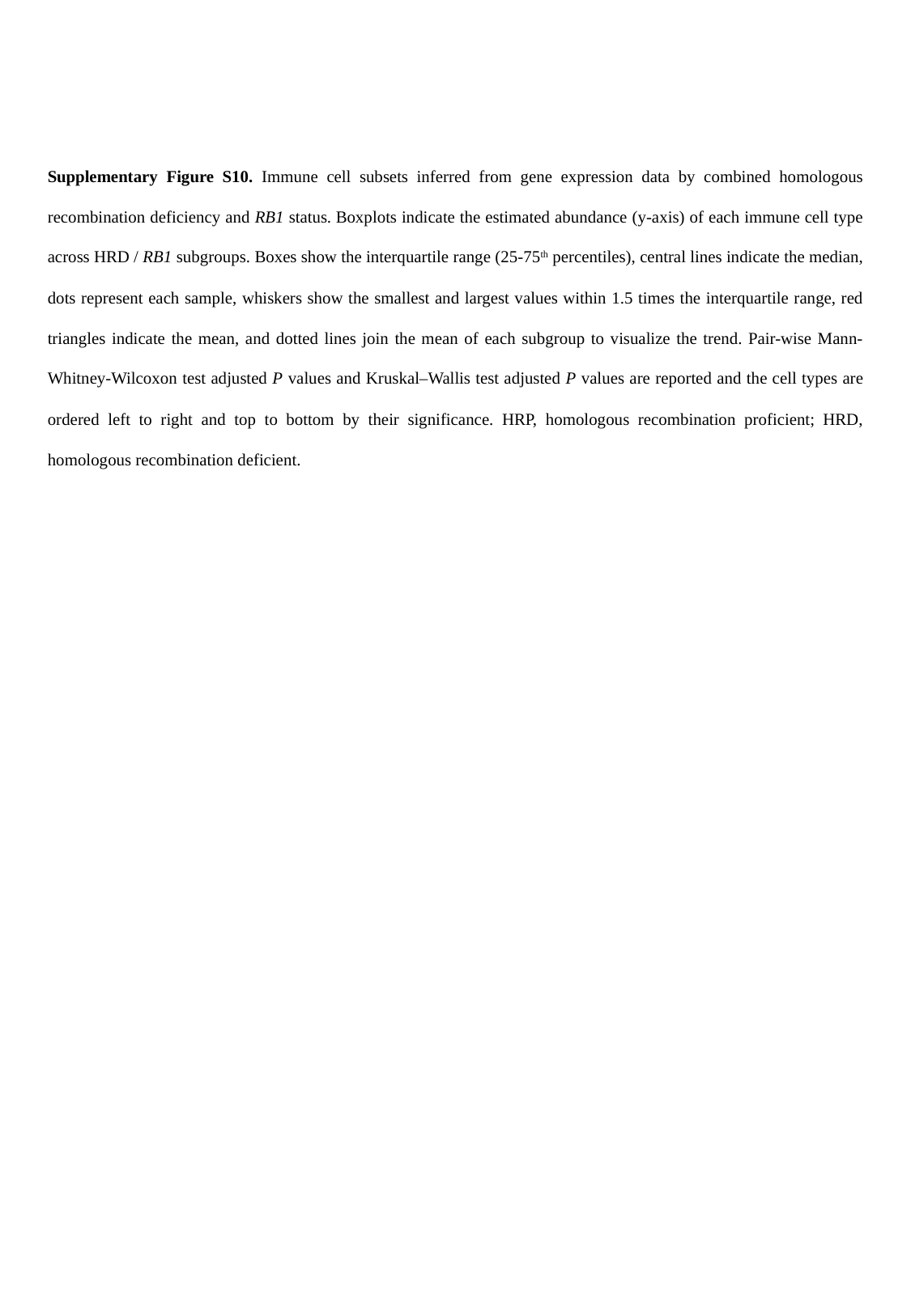

Supplementary Figure S10. Immune cell subsets inferred from gene expression data by combined homologous recombination deficiency and RB1 status. Boxplots ﻿indicate the estimated abundance (y-axis) of each immune cell type across HRD / RB1 subgroups. Boxes show the interquartile range (25-75th percentiles), central lines indicate the median, dots represent each sample, whiskers show the smallest and largest values within 1.5 times the interquartile range, red triangles indicate the mean, and dotted lines join the mean of each subgroup to visualize the trend. Pair-wise Mann-Whitney-Wilcoxon test adjusted P values and Kruskal–Wallis test adjusted P values are reported and the cell types are ordered left to right and top to bottom by their significance. HRP, homologous recombination proficient; HRD, homologous recombination deficient.
